# Target and Non-Target Analysis of Polycyclic Aromatic Hydrocarbons and Emerging Aromatic Contaminants in Outdoor Dust from a Petrochemical-Impacted Residential Area

**DOI:** 10.3390/toxics14030223

**Published:** 2026-03-05

**Authors:** Yimeng Si, Siyuan Li, Yu Wang, Hao Chen, Yanlong Zhang, Shaoping Kuang, Hongwen Sun

**Affiliations:** 1 College of Environment and Safety Engineering, Qingdao University of Science and Technology, Qingdao 266042, China; 4023101041@mails.qust.edu.cn; 2 MOE Key Laboratory of Pollution Processes and Environmental Criteria, College of Environmental Science and Engineering, Nankai University, Tianjin 300350, China; lisiyuan@mail.nankai.edu.cn (S.L.); chenhao@nankai.edu.cn (H.C.); zhangyanlong@nankai.edu.cn (Y.Z.); sunhongwen@nankai.edu.cn (H.S.); 3 Guangdong-Hong Kong-Macao Greater Bay Area Environmental Technology Research Center, Shenzhen Research Institute of Nankai University, Shenzhen 518063, China

**Keywords:** PAH, PAH derivatives, incremental lifetime cancer risk, non-target analysis

## Abstract

The complex contamination characteristics and potential health risks of polycyclic aromatic hydrocarbons (PAHs) and their derivatives remain poorly understood. In this study, a comprehensive analysis of 16 parent PAHs and 34 derivatives was conducted in outdoor dust samples collected from a residential area constructed on an abandoned petrochemical site. The results showed that the total concentrations of PAHs, oxidized PAHs, nitro-PAHs, brominated PAHs, and chlorinated PAHs were in the ranges of 75.3–991 ng/g, 9.27–142 ng/g, 1.68–265 ng/g, 15.2–100 ng/g, and 1.23–14.8 ng/g, respectively. Additionally, the non-target screening analysis identified 29 potential aromatic compounds in dust samples. Toxicity assessment indicated that several PAH derivatives and newly identified compounds exhibited stronger acute toxicity than PAHs (ECOSAR model prediction). Incremental lifetime cancer risk (ILCR) values of target compounds ranged from 1.54 × 10^−7^ to 2.95 × 10^−6^ for adults and from 5.08 × 10^−8^ to 9.75 × 10^−7^ for children. Oral ingestion was identified as the dominant exposure pathway, accounting for 83.5% of total exposure, followed by dermal contact (16.5%). Overall, these findings highlight the complexity of human exposure to PAHs and related aromatic contaminants in petrochemical-impacted residential areas and underscore the need for continued attention to their associated environmental and health risks.

## 1. Introduction

Polycyclic aromatic hydrocarbons (PAHs) are a class of persistent organic pollutants (POPs) composed of two or more benzene rings arranged in a polycyclic structure. They are widely distributed across various environmental media, including the atmosphere [1,2], water bodies [3,4], soil [5], and sediments [6]. Many PAHs exhibit strong carcinogenic properties, leading the U.S. Environmental Protection Agency (USEPA) to designate 16 PAHs as contaminants of concern [7]. PAH derivatives are formed when PAHs undergo various environmental processes, including photochemical reactions, microbial degradation, and biotransformation, during which hydrogen atoms on the aromatic rings are substituted by functional groups such as hydroxyl, nitro, or halogen groups. Compared with their parent PAHs, these derivatives generally exhibit greater environmental mobility and substantially higher biological toxicity [8].

Petrochemical sites are recognized as significant sources of PAHs and their derivatives. Previous studies conducted at petrochemical sites in Shandong [9], Beijing [10], and Lanzhou [11], China, reported PAH concentrations of 2.43 × 10^3^–13.4 × 10^3^ ng/g and derivative concentrations of 2.12 × 10^3^–4.26 × 10^4^ ng/g, with average levels 1.3 to 27 times higher than those observed in surrounding residential areas. In addition, commercial and residential areas may experience remarkable human exposure to PAHs and their derivatives due to urban traffic emissions and fuel combustion associated with daily activities [12]. In recent years, China has implemented the policy of “retreat from the city and move to the industrial park” for chemical enterprises. As a result, many former petrochemical sites have been remediated and redeveloped into residential or commercial areas. However, the background contamination of PAHs in these redeveloped areas may be more complex, and relevant studies addressing their occurrence and health risks remain limited.

Dust possesses distinct physical properties, including a large specific surface area and strong adsorption capacity, enabling it to readily accumulate PAHs and their derivatives from the environment through hydrophobic interactions and van der Waals forces. Consequently, dust acts as an important environmental carrier and migration medium for these compounds [13]. As a result, dust has been widely recognized as a key indicator for human exposure to PAHs and their derivatives [14,15,16,17,18]. Analyzing PAHs and their derivatives in outdoor settled dust from residential areas can provide valuable insights into local contamination patterns, assess health exposure and associated health risks, and support the development of targeted pollution control and management strategies.

PAHs and their derivatives represent only the most extensively studied subgroup of polycyclic aromatic contaminants (PACs). Gas chromatography coupled with high-resolution mass spectrometry (GC-HRMS) has demonstrated significant advantages for trace analysis and non-target screening, including high-quality accuracy, excellent selectivity, and high sensitivity [19,20,21]. This technique enables reliable qualitative identification of target compounds while effectively minimizing matrix interference. Therefore, establishing a GC-HRMS-based analytical approach allows for a more comprehensive characterization of the complex mixture of PACs present in residential dust.

In this study, outdoor settled dust collected from residential areas developed on a former petrochemical plant site was investigated. The specific objectives were to: (1) characterize the contamination profile of PAHs and their derivatives in outdoor dust; (2) identify additional potential PACs using GC-HRMS-based non-target screening; (3) assess the carcinogenic risks associated with target PAHs and their derivatives.

## 2. Materials and Methods

### 2.1. Chemicals and Reagents

This study investigated 16 parent PAHs and 34 PAH derivatives, including 5 oxygenated PAHs (O-PAHs), 9 nitro-PAHs (N-PAHs), 13 brominated PAHs (Br-PAHs), and 7 chlorinated PAHs (Cl-PAHs), as well as isotope-labeled internal standards corresponding to 16 parent PAHs and 6 derivatives employed for quantitative analysis. Detailed information on target compounds is provided in Tables S1 and S2 of the Supplementary Information (SI). A saturated alkane mixture (C_7_-C_40_) was used to calculate the Kováts retention index (RI) for the identification of potential unknown contaminants during non-target screening. The sources and specifications of reagents, standards and consumables used in this study are described in Text S1.

### 2.2. Sample Collection

Dust samples were collected in November 2024 from a residential area in eastern China that had a long history of petrochemical activities prior to residential development. After industrial operations ceased, the site underwent remediation and was subsequently redeveloped into residential areas. Outdoor settled dust samples were collected from 10 residential locations (Figure S1 and Table S3), specifically from the surface of air-conditioning units, windowsills, and the outer edges of curtains. These surface dusts were originated from airborne particulate aggregation and deposition, as such surfaces are not direct PAH sources and thus reflect generally regional dust intake exposure pathways of these compounds. Dust was collected using clean brushes that were pre-rinsed with anhydrous ethanol, air-dried, and wrapped in aluminum foil prior to use. All collected dust samples were transported to the laboratory, sieved through a 1 mm stainless steel mesh to remove large debris and impurities, and stored at –20 °C until analysis.

### 2.3. Sample Preparation and Instrument Analysis

Sample preparation was adapted from a previous reported method with minor modifications [18]. Briefly, dust samples were extracted using a modified ultrasonic extraction method. For quantitative analysis, hexane/acetone (*v*:*v* = 1:1) was employed as the extraction solvent. The resulting extracts were purified using silica gel column solid-phase extraction and subsequently filtered through a 0.22 μm PTFE membrane before instrument analysis. For non-target screening analysis, methanol, n-hexane/acetone (*v*:*v* = 1:1), and an ethyl acetate solution were used as extraction solvents, respectively. Following extraction, the samples were subjected to high-speed centrifugation to remove fine particulate matter, after which the supernatants were collected for analysis. Both target quantitative analysis and non-target screening were performed using a GC-Orbitrap HRMS system (Trace 1610 gas chromatograph/Orbitrap Exploris GB 10090, Thermo Fisher Scientific™, Rockford, IL, USA), equipped with a TG-5MS capillary column (30 m × 0.25 mm, 0.25 μm, Thermo Fisher Scientific, MA, USA) and an EI ion source [22]. Detailed information regarding sample preparation and instrument procedures is provided in Text S2 and Table S4.

### 2.4. Quality Assurance and Quality Control

For quantitative analysis, calibration curves for all target exhibited good linearity, with a coefficient of determination (R^2^) greater than 0.99. Matrix-spiked recoveries for all compounds ranged from 72.0% to 115%. The instrument limit of quantification (LOQ) was defined as a signal-to-noise ratio (S/N) of 10. For compounds detected in procedural blanks, the method detection limit (MDL) was calculated as three times the standard deviation of the blank concentrations and the sample mass. For compounds not detected in blanks, the MDL was calculated based on the instrumental LOQ and sample volume. The calculated MDLs for target compounds were 0.002–3.49 ng/g. All reported concentrations were corrected by subtraction of procedural blank values. Detailed quality assurance and quality control results are provided in Table S5.

### 2.5. Workflow for Non-Target Analysis

A non-target analysis was conducted to comprehensively characterize the complexity of contamination in dust samples. Raw data obtained from GC-Orbitrap HRMS were deconvoluted using Compound Discoverer (CD, version 3.3, Thermo Fisher Scientific, Rockford, IL, USA) to identify unknown contaminants (Figure S2). The “GC EI with Statistics” workflow in CD software (version 3.3) was optimized and applied for primary peak separation, preliminary library matching, and potential PACs screening (specific parameter settings in CD software are detailed in Table S6). Mass spectra for candidate contaminants were extracted using Qual Browser in Xcalibur 4.6 (Thermo Fisher Scientific, Rockford, IL, USA), followed by refined library matching and fragment ion interpretation using NIST MS Search (version 2.4) and MS Interpreter (version 3.4.4). The retention index (RI), which describes the chromatographic retention behavior of compounds [23], was employed as an additional identification constraint to improve confidence [24,25,26,27]. RI values for candidate contaminants were calculated by analyzing a saturated alkanes standard mixture (C_7_-C_40_) under identical instrumental conditions. To account for the inherent uncertainty associated with RI, arising from both experimental variability and reference data distributions, a mass spectral match was only considered confident if the ΔRI < 20 (confidence level > 95%). Furthermore, based on the previously established confidence level (CL) framework [27], all identified compounds were assigned a confidence level of 3 or higher. Detailed screening procedures and confidence level assessment criteria are provided in Text S3. Matrix effects were assessed by comparing the peak areas of the internal standard in the samples with those in the standard solution, and all were found to be acceptable (74.8–119%, Table S7).

### 2.6. Toxicity and Risk Assessment

The Ecological Structure–Activity Relationship model (ECOSAR V2.2) was employed to predict the acute toxicity of target PAHs, their derivatives, and non-target screening compounds for soil (earthworm) and aquatic organisms (fish). Acute toxicity was expressed as median lethal concentration (LC_50_) values.

Toxic equivalent factors (TEFs) were used to assess the relative carcinogenic toxicity of PAHs [13]. Within the TEF system, benzo[a]pyrene (BaP) is designated as the reference compound with a TEF value of 1, while other PAHs are assigned TEF values based on their carcinogenic potency relative to BaP [28,29,30]. TEF values for 29 target pollutants obtained from studies are summarized in Table S8. Toxic equivalence quantities (TEQs), representing the overall toxicity of PAH mixtures, were calculated using the following equation:

(1) $$\mathrm{TEQs}\;=\;\sum \;C_{i}\;\times \;{\mathrm{TEF}}_{i}$$

where C*_i_* is the measured concentration of each compound *i*, and TEF*_i_* is its corresponding toxic equivalent factors.

The incremental lifetime cancer risk (ILCR), a standardized model developed by the U.S. Environmental Protection Agency (EPA) [31], was used to evaluate the carcinogenic risk associated with exposure to PAHs and their derivatives [32,33]. In this study, ILCRs were estimated for three exposure pathways: ingestion, inhalation, and dermal adsorption. The total carcinogenic risk was obtained by summing the ILCRs from three pathways. The equations used are as follows:

(2) $${\mathrm{ILCR}}_{\mathrm{Ingestion}}=\frac{\mathrm{CS}\times ({\mathrm{CSF}}_{\mathrm{Ingestion}}\times \sqrt[{3}]{\frac{\mathrm{BW}}{70}})\times {\mathrm{IR}}_{\mathrm{Ingestion}}\times \mathrm{EF}\times \mathrm{ED}}{\mathrm{BW}\times \mathrm{AT}\times 10^{6}}$$

(3) $${\mathrm{ILCR}}_{\mathrm{Inhalation}}=\frac{\mathrm{CS}\times ({\mathrm{CSF}}_{\mathrm{Inhalation}}\times \sqrt[{3}]{\frac{\mathrm{BW}}{70}})\times {\mathrm{IR}}_{\mathrm{Inhalation}}\times \mathrm{EF}\times \mathrm{ED}}{\mathrm{BW}\times \mathrm{AT}\times \mathrm{PEF}}$$

(4) $${\mathrm{ILCR}}_{\mathrm{Dermal}}=\frac{\mathrm{CS}\times ({\mathrm{CSF}}_{\mathrm{Dermal}}\times \sqrt[{3}]{\frac{\mathrm{BW}}{70}})\times \mathrm{SA}\times \mathrm{AF}\times \mathrm{ABS}\times \mathrm{EF}\times \mathrm{ED}}{\mathrm{BW}\times \mathrm{AT}\times 10^{6}}$$

(5) $$\mathrm{ILCRs}={\mathrm{ILCR}}_{\mathrm{Ingestion}}+{\mathrm{ILCR}}_{\mathrm{Inhalation}}+{\mathrm{ILCR}}_{\mathrm{Dermal}}$$

The carcinogenic slope factors (CSF) for BaP via ingestion, inhalation, and dermal adsorption are 7.3, 3.85, and 25 mg/kg/day, respectively. Definitions, reference values, and sources for all exposure parameters are provided in Table S9.

According to carcinogenic risk assessment guidelines established by the U.S. EPA [31] and the World Health Organization (WHO) [34], an ILCR ≤ 10^−6^ indicates an acceptable level. An ILCR of 10^−6^ ≤ ILCR ≤ 10^−4^ is considered a “tolerable risk range,” warranting further monitoring or management, whereas an ILCR > 10^−4^ indicates an unacceptable high-risk level, requiring immediate remediation.

### 2.7. Data Analysis and Statistics

Quantitative analysis of target compounds was performed using the Quan Browser software (Xcalibur 4.6, Thermo Fisher Scientific Inc., San Jose, CA, USA). Spearman’s rank correlation analysis was conducted using IBM SPSS Statistics 26 (IBM Corporation, Armonk, NY, USA). Data visualization was performed using Origin 2021 (Origin Lab Corporation, Northampton, MA, USA) and ArcMap 10.8 (ESRI Inc., Redlands, CA, USA) software. Chemical structures were drawn using ChemDraw 20.0 (PerkinElmer Informatics, Waltham, MA, USA).

## 3. Results and Discussion

### 3.1. Concentrations of Target PAHs and Their Derivatives

**Figure 1:**
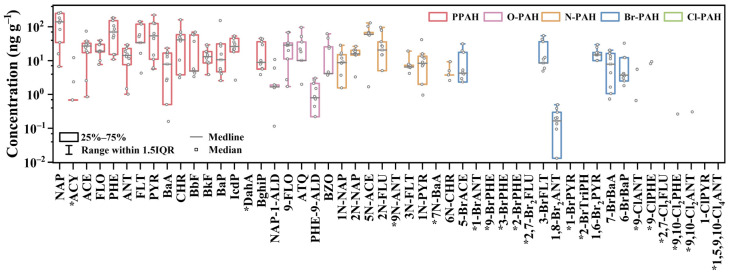
Concentration of PAHs and their derivatives in outdoor dust from a petrochemical-impacted residential area. ( *: Lines not shown in the figure indicate a median value not detected.)

A total of 37 target pollutants were detected in the outdoor dust samples, including 15 PAHs, 5 O-PAHs, 7 N-PAHs, 6 Br-PAHs, and 4 Cl-PAHs (Figure 1). Among these, naphthalene (NAP), acenaphthene (ACE), fluorene (FLO), phenanthrene (PHE), anthracene (ANT), pyrene (PYR), benzo[k]fluoranthene (BkF), and benzo[a]pyrene (BaP), and 9-fluorenone (9-FLO), were detected in 100% of the samples. The total concentrations of PAHs (∑PAHs), O-PAHs (∑O-PAHs), N-PAHs (∑N-PAHs), Br-PAHs (∑Br-PAHs), and Cl-PAHs (∑Cl-PAHs) were in the ranges of 75.3–991 ng/g, 9.27–142 ng/g, 1.68–265 ng/g, 15.2–100 ng/g, and 1.23–14.8 ng/g (Table S10). The corresponding median concentrations were 721 ng/g for ∑PAHs, 69.6 ng/g for ∑O-PAHs, 122 ng/g for ∑N-PAHs, 54 ng/g for ∑Br-PAHs, and 8.03 ng/g for ∑Cl-PAHs. Overall, PAH concentrations in this study were lower than those reported for residential areas in previous studies (0.07 to 345 μg/g) [14,16,35]. Considerable variability was observed among individual PAHs, with NAP exhibiting the highest concentration (total: 1360 ng/g, median: 140 ng/g), consistent with patterns reported in previously studies [35,36]. Among the PAH derivatives, N-PAHs exhibited a relatively broad concentration range and the highest median concentration. Previous studies have also reported elevated N-PAH concentrations in outdoor environmental dust [13], and are likely attributable to enhanced atmospheric photochemical reactions under strong solar radiation, which promote the secondary formation of nitro-substituted PAHs [37,38].

**Figure 2:**
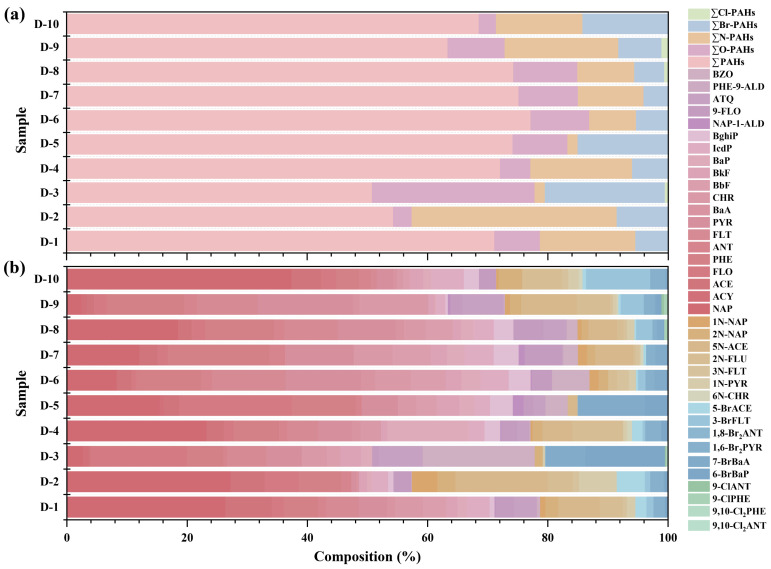
Compositions of PAHs and their derivatives in outdoor dust from a petrochemical-impacted residential area: (**a**) distribution of PAHs, O-PAHs, N-PAHs, Br-PAHs, and Cl-PAHs; (**b**) distribution of individual contaminants.

As shown in Figure 2a, parent PAHs were the dominant pollutants in the residential dust samples, accounting for 50.8% to 77.2% of total target compounds. At sampling sites D-1, D-2, D-5, and D-10, low-molecular-weight PAHs (2–3 rings) were predominant, contributing 43.2%, 47.4%, 49.1%, and 51% of total PAHs, respectively. In contrast, sites D-6 and D-9 exhibited higher proportions of high-molecular-weight PAHs (4–6 rings), accounting for 53.0% and 41.8% of total PAHs, respectively (Figure 2b). At sites D-2 and D-3, PAH derivatives constituted substantial proportions of the total pollutants, accounting for 45.6% and 49.2%, respectively. At site D-3, O-PAHs (29.1%) and Br-PAHs (19.9%) were the dominant derivative classes, with benzanthrone (BZO: 18.6%) and 6-bromobenzo [a]pyrene (6-BrPYR: 13.2%) as the major contributors. Previous studies have indicated that O-PAHs and parent PAHs often share common sources, including incomplete fuel combustion [39] and non-exhaust emissions [16]. The elevated proportion of Br-PAHs at this site may be associated with emissions from electronic-waste-related activities [40]. At site D-2, the relative contribution of N-PAHs was notably higher (34.1%), with 5-nitroacenaphthene (5N-ACE) accounting for 15.4% of total derivatives. This pattern is likely attributed to the site’s proximity to a roadway, resulting in increased exposure to vehicular emissions [41]. Overall, consistent with findings from previous studies [42,43], the diversity of PAHs and their derivatives may indicate multiple pollution sources at this site, exploratorily highlighting the complex pollution patterns present in residential areas built on former petrochemical plant sites.

### 3.2. Spearman’s Correlation Analysis of Target PAHs and Derivatives

**Figure 3:**
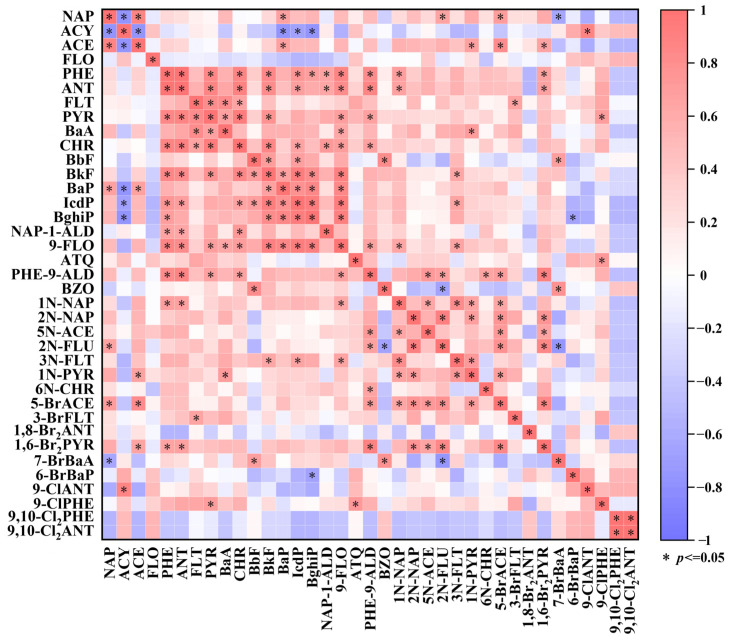
Spearman’s correlation analysis of PPAH and derivative concentrations (red: positive correlation; blue: negative correlation; asterisks *: statistical significance *p* ≤ 0.05).

To further elucidate the relationships between parent PAH and their derivatives, Spearman’s rank correlation analysis was performed on the concentrations of target pollutants (Figure 3). Strong positive correlations were observed among several parent PAHs, including PHE, ANT, FLT, PYR, benzo[a]anthracene (BaA), chrysene (CHR), benzo[b]fluoranthene (BbF), BkF, BaP, and indeno [1,2,3-c,d]pyrene (IcdP). For example, significant correlations were found between ANT and PHE (r = 0.99, *p* < 0.05), PYR and CHR (r = 0.94, *p* < 0.05), and BkF and IcdP (r = 0.93, *p* < 0.05), indicating that these PAHs may exhibit similar environmental transport and fate behaviors. Several derivatives, including naphthalene-1-aldehyde (NAP-1-ALD) and 9-FLO, also showed strong positive correlations with the above parent PAHs. This suggests that these derivatives may be formed via secondary transformation processes of parent PAHs, such as radical-mediated reactions [44]. Additionally, 1,6-Br_2_PYR exhibited a significant positive correlation with phenanthrene-9-aldehyde (PHE-9-ALD) (r = 0.89, *p* < 0.05), while 5N-ACE was positively correlated with 5-bromoacenaphthene (5-BrACE) (r = 0.73, *p* < 0.05). Previous research has similarly reported a shared source of O-PAHs and Br-PAHs [45]. In contrast, although Br-PAH and Cl-PAH have often been reported to exhibit strong correlations in environmental matrices [46,47], Cl-PAHs in this study predominantly showed weak or negative correlations with other compounds and were present at relatively low concentrations; this pattern may reflect differences in source inputs, environmental behavior, and transformation or degradation pathways. For instance, many Cl-PAH derivatives are associated with contaminated wastewater discharges [48] or are formed as byproducts of chlorination during water disinfection processes [45]. In the present study, area, redevelopment activities involved minimal wastewater discharge, which may explain the limited occurrence and distinct correlation patterns of chlorinated PAHs.

### 3.3. Identification of Unknown PACs

**Figure 4:**
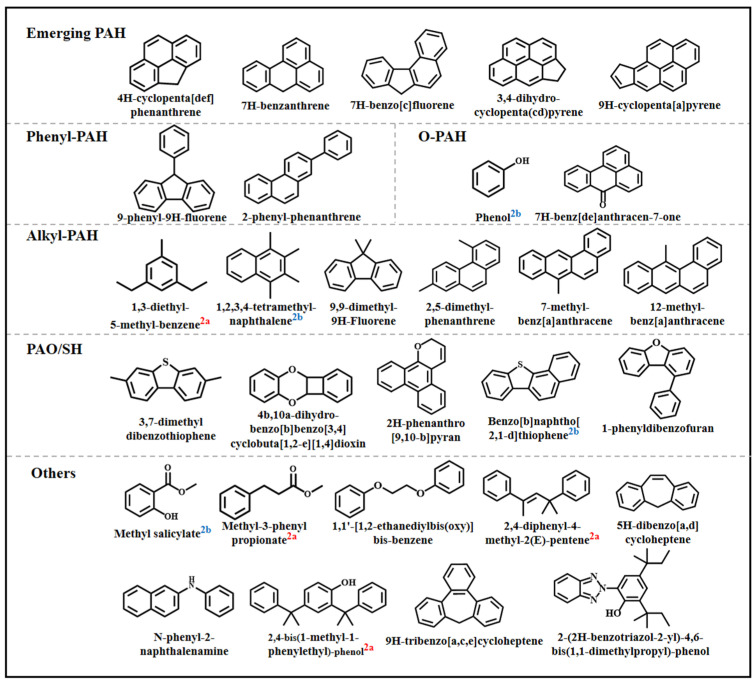
Identified aromatic compounds by non-target screening analysis (compound labeled with 2a and 2b represent confidence levels of “Level 2a and 2b”; unlabeled compounds have confidence level of “Level 3”).

The contamination profiles of PAHs and their derivatives observed in this study highlight the substantial complexity of PACs present in residential dust. Previous studies have indicated that 16 priority PAHs account for less than 20% of the total equivalent levels (TEQs) of PAHs and their derivatives [9,49,50], underscoring the importance of identifying additional PACs beyond the commonly monitored compounds. Therefore, non-target screening using GC-HRMS was employed to further characterize potential PACs in dust samples. Following data acquisition, raw data files were processed using CD software. Initial spectra deconvolution and library matching of 307,869 spectra resulted in 3971 detected compound peaks. To reduce false positives, only peaks with peak areas ≥10 times higher than those observed in procedure blank peak and search indices ≥600 were retained, yielding 251 candidate compounds. Among these, 95 compounds were preliminarily assigned as aromatic based on accurate mass matching (mass error <10 ppm). For these 95 aromatic candidates, further spectral confirmation was conducted using library matching and fragment ion interpretation with NIST MS Search and MS Interpreter tools in Xcalibur software (version 4.6). By integrating the results from multi-software platforms and applying stringent identification criteria, a total of 29 aromatic pollutants were ultimately identified (Table S11). According to the confidence level criteria, 4 compounds were classified as Level 2a (Spectrum matches library, and the calculated ΔRI < 20), 4 as Level 2b (ΔRI < 20, incomplete mass spectra and fragment evidence), and 21 as Level 3 (Search index ≥ 600, mass error < 5 ppm, incomplete mass spectra and fragment evidence, no RI recorded in the database or ΔRI > 20). Based on functional group classification, the newly identified compounds included five emerging PAHs, two phenyl-PAHs, 2 O-PAHs, six alkyl-PAHs, three oxygenated polycyclic aromatic hydrocarbons (PAOHs), and two polycyclic aromatic sulfur heterocycles (PASHs). The remaining compounds were grouped into an “Others” category (Figure 4).

Literature searches for the newly identified compounds were conducted using SciFinder and PubChem. The results indicated that 3 compounds were tentatively identified as potential new contaminants in environmental media, while 11 compounds were tentatively identified in dust. The presence of these compounds in their respective matrices has not been documented in existing literature.

All five detected emerging PAHs were classified as Level 3. Among them, 7H-benzanthrene (Figure S2-2), 3,4-dihydro-cyclopenta(cd)pyrene (Figure S2-4), and 9H-cyclopenta[a]pyrene (Figure S2-5) have been reported in atmospheric particulates [51,52] and aerosols [53], but not in dust-related literature. The presence of 7H-benzanthrene suggests potential biomass burning in nearby residential areas [53]. Historically, studies on phenyl-PAHs have largely focused on their associations with petrochemical activities interactions [54]. In this study, the presence of phenyl-PAHs such as 9-phenyl-9H-fluorene (Figure S2-6) and 2-phenyl-phenanthrene (Figure S2-7) further suggests that the study area remains influenced by legacy petrochemical production activities.

A total of six alkyl-PAHs were detected in dust samples, among which 1,3-diethyl-5-methyl-benzene (Figure S2-10) and 12-methyl-benz[a]anthracene (Figure S2-15), which had not previously been reported in dust. 1,3-diethyl-5-methyl-benzene is classified as a Group 2A carcinogen and has been quantified in industrial exhaust emissions at concentrations ranging from 0.32 to 0.88 mg m^−3^ [55]. Additionally, 1,2,3,4-tetramethylnaphthalene (Figure S2-11), classified as Group 2B, has been detected in tobacco leaves [56] and tobacco smoke [57]. Functional group substitutions can substantially alter the physicochemical properties of PAHs and often enhance their toxicological potency, with methylation generally associated with increased toxicity [58,59]. For example, 12-methyl-benz[a]anthracene adsorbed onto airborne particulate matter has been shown to induce non-genetic carcinogenic effects [60]. Similarly, 2,5-dimethyl-phenanthrene (Figure S2-13) has been detected in coal mine fire zones at concentrations far exceeding those of 16 PAHs [61]. Moreover, 9,9-dimethyl-9H-fluorene (Figure S2-12) has been detected in tobacco and cannabis smoke and has been associated with carcinogenic, mutagenic, and teratogenic effects [57].

Furthermore, heterocyclic aromatic pollutants containing nitrogen, sulfur, and oxygen are persistent contaminants commonly found in fuels and emissions [62,63]. In this study, a total of five PAO/SHs were identified. Among them, 4b,10a-dihydro-Benzo[b]benzo [3,4]cyclobuta [1,2-e][1,4]dioxin (Figure S2-17) has not been previously reported in environmental media; this compound has previously been reported only as an intermediate in photo-oxidative organic synthesis [64]. In addition, 2H-phenanthro [9,10-b]pyran (Figure S2-18), 1-phenyldibenzofuran (Figure S2-20), and 3,7-dimethyldibenzothiophene (Figure S2-16) were also not previously reported in dust.

Among the nine compounds classified as “Others”, 5H-dibenzo[a,d]cycloheptene (Figure S2-25) and 9H-tribenzo[a,c,e]cycloheptene (Figure S2-28) have been frequently reported as intermediates in catalytic synthesis [65] and pyrolysis reactions [66], respectively, and are herein reported in environmental samples. Methyl-3-phenylpropionate (Figure S2-22) and 2,4-diphenyl-4-methyl-2(E)-pentene (Figure S2-24) were classified as Level 2a, while methyl salicylate was classified as Level 2b. These three compounds were detected in dust for the first time.

It should be noted that most compounds identified through non-target screening in this study are provisionally classified as Level 3 results and were not confirmed using authentic reference standards; therefore, structural isomers cannot be excluded. Accordingly, the interpretation of these tentatively identified compounds should be approached with caution.

### 3.4. Risk Assessment

**Figure 5:**
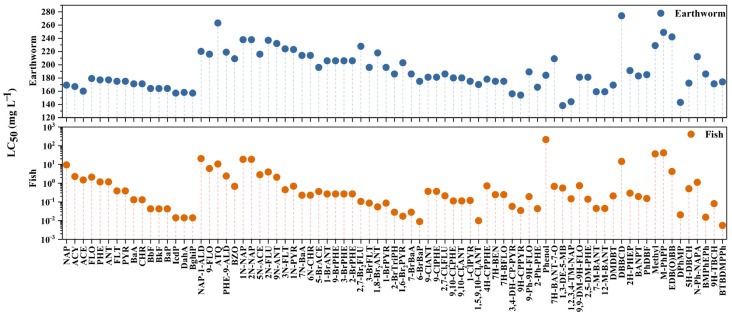
The LC_50_ (mg/L) of target and screening PACs for soil organisms (earthworm) and aquatic organisms (fish).

The acute toxicity of target pollutants and screened compounds to soil and aquatic organisms was predicted using ECOSAR V2.2. LC_50_ (mg/L) was estimated for earthworm (14d) and fish (96h), with the results summarized in Table S12 and Figure 5. For all analyzed compounds, the LC_50_ values ranged from 138 to 274 mg/L for earthworms and from 0.0055 to 212 mg/L for fish. Model predictions indicated that several PAH derivatives and screened compounds exhibited markedly higher acute toxicity than their corresponding parent PAHs. Alkyl-substituted compounds showed enhanced acute toxicity toward earthworms, exemplified by 1,3-diethyl-5-methylbenzene (1,3-DE-5-MB, LC_50_ = 138 mg/L) and 2,4-diphenyl-4-methyl-2(E)-pentene (DPhMP, LC_50_ = 143 mg/L). For aquatic organisms, 2-(2H-benzotriazol-2-yl)-4,6-bis(1,1-dimethylpropyl)-phenol (BTBDMPPh) (Figure S2-29) exhibited the highest predicted acute toxicity to fish (LC_50_ = 0.0055 mg/L). In addition, halogen substitution was found to substantially increase the acute toxicity of PAHs to aquatic organisms, consistent with previous reports [67]. For instance, 6-bromobenzo[a]pyrene (6-BrBaP), and 1,5,9,10-tetrachloroanthracene (1,5,9,10-Cl_4_ANT) exhibited very low LC_50_ values of 0.011 and 0.013 mg L^−1^, respectively.

Additionally, the carcinogenic risks associated with quantitated PAHs and their derivatives in dust samples were assessed by TEQs of target pollutants using the obtained TEFs (Table S13). Across all sampling sites, total TEQs ranged from 13.2 ng/g to 253 ng/g (median: 47.2 ng/g; mean: 66.6 ng/g). Consistent with previous studies, the seven carcinogenic PAHs defined by the U.S. EPA dominated the total TEQs (BaA: 1.38%; CHR: 0.65%; BbF: 3.55%; BkF: 2.22%; BaP: 41.7%; IcdP: 4.14%) [68,69,70]. Notably, several PAH derivatives also made substantial contributions, including 1-nitropyrene (1N-PYR: 1.64%), 6-nitrochrysene (6N-CHR: 30.9%), and 7-bromobenz[a]anthracene (7-BrBaA: 11.1%). Their elevated contributions are partly attributable to higher TEF values [71] and may also reflect the nitration of parent PAHs in the environment [72].

The ILCR analysis results are presented in Table S14. Total ILCR values for target pollutants ranged from 1.54 × 10^−7^ to 2.95 × 10^−6^ for adults and ranged from 5.08 × 10^−8^ to 9.75 × 10^−7^ for children. All estimated ILCR values for children were below the commonly accepted threshold of 10^−6^, indicating an acceptable carcinogenic risk level under studied conditions. However, age-dependent factors such as inhalation rates and skin exposure areas may influence exposure and carcinogenic risk as individuals grow older [73]. For adults, the lifetime carcinogenic risks associated with dust-borne target pollutants exhibit clear differences among exposure pathways. When all target pollutants were considered collectively, oral ingestion was the dominant exposure pathway, accounting for 83.5% of the total ILCR, followed by dermal contact (16.5%). PAHs contributed the highest proportion to the total ILCR (maximum: 1.85 × 10^−6^; 62.6%), followed by N-PAHs (maximum: 1.10 × 10^−6^; 37.3%), in agreement with previous studies reporting non-negligible carcinogenic risk for N-PAHs [74]. Although the estimated carcinogenic risks of other PAH derivatives remained within acceptable limits, urban environments expose populations to diverse and overlapping emission sources, potentially elevating long-term cancer risks [75]. Therefore, a more comprehensive carcinogenic risk assessment that incorporates a broader range of PACs remains essential.

## 4. Conclusions

This study investigated outdoor dust collected from a residential area constructed on a former petrochemical site. Parent PAHs and their derivatives of O-PAHs, N-PAHs, Br-PAHs, and Cl-PAHs were found at a considerable level in studied area. By using non-target screening techniques, 29 additional aromatic compounds were identified, highlighting the considerable compositional complexity of PACs in outdoor dust. Importantly, the ECOSAR model prediction results indicate that several PAH derivatives and screened PACs exhibited stronger acute toxicity than PAHs. Further, ILCR assessment quantified the carcinogenic risk associated with PAHs and their derivatives through multiple exposure pathways, underscoring the importance of implementing preventive monitoring and expanding toxicological validation measures in the redevelopment of residential areas.

It should be noted that the limited number of sampling sites and the absence of seasonal variation analysis constrain the statistical generalizability of the present findings. Additionally, the compounds identified through non-target screening were not quantitatively analyzed, which may limit a comprehensive evaluation of their relative contributions to overall exposure levels and associated health risk. The ECOSAR toxicity predictions applied in this study are based on structural analogies. The incomplete or variable TEFs values and application domain limits of ECOSAR model may introduce uncertainty into the risk assessment.

Nevertheless, the integration of targeted quantification and non-target screening within well-defined study context enables an in-depth characterization of PAH contamination complexity and provides valuable methodological insights for investigations in historically contaminated residential areas. Future research should further validate the temporal and spatial distribution patterns of newly identified compounds, experimentally confirm their structures, and elucidate their toxicological effects. Moreover, greater emphasis should be placed on assessing the potential risks associated with combined exposure to complex mixtures of PACs.

## Data Availability

The original contributions presented in this study are included in this article/Supplementary Materials. Further inquiries can be directed to the corresponding authors.

## Supplementary Materials

The following supporting information can be downloaded at: https://www.mdpi.com/article/10.3390/toxics14030223/s1. Text S1. Sources of laboratory consumables and reagents; Text S2. Sample preparation and instrument analysis; Text S3. Workflow for non-target screening; Table S1. Chemical properties of target PAHs and their derivatives; Table S2. Basic information on internal standards used in the study; Table S3. Sampling sites information; Table S4. Instrumental parameters of GC-HRMS; Table S5. Procedure blanks, matrix spiked recoveries, and method detection limits (MDLs) of target PAHs and derivatives; Table S6. Detail parameters of GC EI with Statistics; Table S7. Evaluation of Matrix Effects (ME) in Non-Targeted Screening; Table S8. Literature reference values for TEFs used in TEQs calculation; Table S9. Parameters and references required for calculation of incremental lifetime cancer risk (ILCR); Table S10. Concentrations and detection frequency (DF) of target PAHs and their derivatives in dust samples; Table S11. Twenty-nine PACs identified by GC-Orbitrap-HRMS non-target screening analysis; Table S12. The LC_50_ (mg/L) of target and screening abbreviations for soil organisms (earthworm) and aquatic organisms (fish); Table S13. TEQs for target contaminants; Table S14. The results of ILCR analysis; Figure S1. The sampling sites distribution; Figure S2. Chromatogram (left) and mass spectrum (right) of 29 identified PACs. References [76,77,78,79,80,81,82,83,84] are citied in the Supplementary Materials.
