# Supplementary material for: Target and Non-Target Analysis of Polycyclic Aromatic Hydrocarbons and Emerging Aromatic Contaminants in Outdoor Dust from a Petrochemical-Impacted Residential Area
Source: Toxics. 2026 Mar 5;14(3):223. doi: 10.3390/toxics14030223 (PMC13029854; doi:10.3390/toxics14030223)
Supplement: Supplementary file 1 [file toxics-14-00223-s001.zip › toxics-4155753-supplementary.pdf]

Supplementary Information

Target and Non-Target Analysis of Polycyclic Aromatic Hydrocarbons and Emerging Aromatic Contaminants in Outdoor Dust from a Petrochemical-Impacted Residential Area

Yimeng Si [a, b], Siyuan Li [b], Yu Wang [b, c, *], Hao Chen [b], Yanlong Zhang [b], Shaoping Kuang [a, *], Hongwen Sun [b]

[a] College of Environment and Safety Engineering, Qingdao University of Science and Technology, Qingdao 266042, China

[b] MOE Key Laboratory of Pollution Processes and Environmental Criteria, College of Environmental Science and Engineering, Nankai University, Tianjin 300350, China

[c] Guangdong-Hong Kong-Macao Greater Bay Area Environmental Technology Research Center, Shenzhen Research Institute of Nankai University, Shenzhen 518063, China

*Corresponding Authors:

Y. Wang (yu.wang@nankai.edu.cn) and S. P. Kuang (spkuang@qust.edu.cn)

Total pages: 42

Number of tables: 14

Number of figures: 2

Contents

Text S1. Sources of laboratory consumables and reagents 3
Text S2. Sample preparation and instrument analysis 3
Text S3. Workflow for non-target screening 4
Table S1. Chemical properties of target PAHs and their derivatives 6
Table S2. Basic information on internal standards used in the study. 14
Table S3. Sampling sites information 14
Table S4. Instrumental parameters of GC-HRMS 15
Table S5. Procedure blanks, matrix spiked recoveries, and method detection limits (MDLs) of target PAHs and derivatives 16
Table S6. Detail parameters of *GC EI with Statistics*. 18
Table S7. Evaluation of Matrix Effects (ME) in Non-Targeted Screening 19
Table S8. Literature reference values for TEFs used in TEQs calculation. 20
Table S9. Parameters and references required for calculation of incremental lifetime cancer risk (ILCR). 21
Table S10. Concentrations and detection frequency (DF) of target PAHs and their derivatives in dust samples. 22
Table S11. Twenty-nine PACs identified by GC-Orbitrap-HRMS non-target screening analysis 24
Table S12. The LC_{50} (mg/L) of target and screening abbreviations for soil organisms (earthworm) and aquatic organisms (fish). 28
Table S13. TEQs for target contaminants. 32
Table S14. The results of ILCR analysis. 34
Figure S1. The sampling sites distribution. 35
Figure S2. Chromatogram (left) and mass spectrum (right) of 29 identified PACs. 36
References 42

Text S1. Sources of laboratory consumables and reagents

The mixed standard for the 16 parent PAHs was purchased from Manji Biotechnology Co., Ltd. (Beijing, China), while the mixed surrogate internal standard and individual standards for various derivatives were obtained from ANPEL Laboratory Technology Inc. (Shanghai, China). Solid-phase extraction (SPE) was performed using CNWBOND Si columns (1 g, 6 mL) purchased from ANPEL Laboratory Technology Co., Ltd. (Shanghai, China). Hydrophobic PTFE needle filters were obtained from Ampere Laboratory Supplies (Shanghai, China). HPLC-grade solvents (hexane, dichloromethane, and methanol) were supplied by ANPEL Laboratory Technology Co., Ltd. (Shanghai, China), while HPLC-grade acetone was purchased from Kangde Technology Co., Ltd. (Tianjin, China). Deionized water was prepared using the Milli-Q Reference water purification system from Merck KGaA (Darmstadt, HE, Germany).

Text S2. Sample preparation and instrument analysis

Quantitative analysis:

An aliquot of 100 mg dust sample was transferred into a 40 mL EPA vial, followed by the addition of mixed internal standards (20 ppb). Subsequently, 10 mL of hexane–acetone (v:v = 1:1) was added. The mixture was ultrasonicated at 25 °C for 30 min at full power and then centrifuged at 2860 g for 5 min. The supernatant was collected, and the extraction procedure was repeated once. The combined extracts were concentrated to approximately 1 mL under a gentle nitrogen stream. Target compounds were separated and enriched using solid-phase extraction (SPE). A silica gel mini-column was preconditioned with 5 mL dichloromethane and equilibrated twice with 5 mL n-hexane. The concentrated extract was loaded onto the column, followed by two elutions with 2 mL n-hexane and two elutions with n-hexane–dichloromethane (v:v = 1:1). The combined eluate was evaporated to near dryness under nitrogen and reconstituted in 1 mL n-hexane. The final solution was filtered through a 0.22 μm PTFE membrane, transferred to an autosampler vial, and stored at −20 °C prior to instrumental analysis.

Non-target screening analysis:

For non-target screening, sequential extraction was performed using solvents of increasing polarity: 10 mL methanol, 10 mL hexane–acetone (v:v = 1:1), and 10 mL ethyl acetate. Each extraction involved shaking at 1800 rpm for 10 min, ultrasonication at 25 °C for 30 min, and centrifugation at 2860 g for 5 min. The combined extracts (30 mL) were further clarified by high-speed centrifugation at 4 °C and 13,000 rpm for 10 min to remove fine particulates. The supernatant was transferred to a sample vial using a glass pipette and stored at −20 °C for subsequent analysis.

Instrumental analysis:

Chemical separation was performed using a TG-5MS capillary column (30 m × 0.25 mm, 0.25 μm, Thermo Fisher Scientific, MA, USA) installed on a GC-Orbitrap HRMS system (Trace 1610 gas chromatograph/Orbitrap Exploris GB 10090, Thermo Fisher ScientificTM). Samples were injected in splitless mode using a 2 μL injection volume, with a carrier gas flow rate of 1.0 mL/min and an injection port temperature of 260 °C. The GC oven temperature program was as follows: initial temperature of 40 °C, held for 1.5 min; Ramp at 25 °C/min to 90 °C, held for 1.5 min; Ramp at 25 °C/min to 180 °C; Ramp at 5 °C/min to 280 °C, held for 1.5 min; Final ramp at 10 °C/min to 310 °C, held for 10 min; Total runtime of 44.60 min. The transfer line temperature was set at 280 °C, ion source temperature at 250 °C, with the electron energy of 70 eV and emission current of 50 μA. The instrument was operated in full scan mode over a mass range of m/z 50–750, with a mass resolution of 30,000. The automatic gain control was set to "Standard," and the maximum ion injection time was set to "AUTO".

Text S3. Workflow for non-target screening

Import the raw measured data into Compound Discoverer software and apply its built-in "GC EI with Statistics" workflow after minor adjustments. The screening process for unknown compounds is as follows:

(1) Set preliminary screening parameters for unknown peaks: mass error < 10 ppm, signal-to-noise ratio (S/N) > 3, peak smoothing points > 9, minimum total ion current (TIC) > 10^6, and ion overlap window > 95%.

(2) Align suspect peaks across different samples based on spectrum dot product and retention times, with a maximum retention time deviation of 5 seconds and a minimum dot product threshold of 500.

(3) After preliminary separation of unknown peaks, perform initial substance identification using databases including the National Institute of Standards and Technology (NIST) "mainlib" and "replib," along with two in-house GC-Orbitrap databases ("gc-orbitrap contaminants library v1.5" and "gc-orbitrap other environmental"). Retain substances with a search index greater than 600.

(4) After subtracting background noise peaks, peak areas were normalized using the "Constant Median" algorithm, and missing values were imputed using the "Median + Small Value with Variability" algorithm.

(5) Retain peaks with a sample-to-blank ratio greater than 5 as candidate peaks. Based on the preliminary structural predictions from matches, the final exported substance list includes only compounds exhibiting aromatic structural features. Record their retention times, m/z values, provisional molecular formulas, and names.

(6) Spectral and structural confirmation of candidate compounds was performed using Qual Browser in Xcalibur 4.6 software, along with NIST MS Search and MS Interpreter. Qual Browser extracted compound spectra from raw data; NIST MS Search performed database spectral matching, retaining candidates with the highest "Match" and "R.Match" scores and minimal deviation; MS Interpreter conducted fragment prediction and structural interpretation.

Based on the CL evaluation framework with adjustments, the final confidence levels for pollutant identification are defined as follows:

CL=3: Search index ⩾ 600, mass error < 5 ppm, incomplete mass spectra and fragment evidence, no RI recorded in the database or △RI > 20;

CL=2b: △RI < 20, incomplete mass spectra and fragment evidence;

CL=2a: Spectrum matches library, and the calculated △RI < 20.

The calculation formula for Kováts RI is as follows:

$$RI = 100[z + \frac{log_{10}RT(x) - log_{10}RT(z)}{log_{10}RT(z+1) - log_{10}RT(z)}]$$

where RI is the retention index of unknown compound x, z is the number of carbon atoms in the n-alkane eluted before unknown compound x, z + 1 is the number of carbon atoms in the n-alkane eluted after unknown compound x, and RT(x), RT(z), and RT(z+1) are the retention times corresponding to the substances, respectively.

Table S1. Chemical properties of target PAHs and their derivatives

**Chemicals**	**Abbreviation**	**CAS#**	**Formula**	**M.W.**	**Structure**	**Solubility (mg L^{-1}) 25 °C**	**Vapor pressure (mmHg) 25 °C**	**Henry's law constant (atm/m^3/mol) 25 °C**	**log K_{OW}**	**log K_{OC}**	**log K_{OA}**	**Half-life time (h) (Level III Fugacity Model; Air/Water/Soil/Sediment)**	**BCF (L kg^{-1})**
						**PAHs**							
Naphthalene	NAP	91-20-3	$C_{10}H_8$	128.17	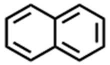	142	8.50E-2	4.40E-4	3.30	2.86	5.19	11.9/900/1.8E3/8.1E3	69.9
Acenaphthylene	ACY	208-96-8	$C_{12}H_8$	152.19	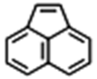	2.49	6.68E-3	1.14E-4	3.94	3.42	6.27	0.744/360/720/3.24E3	185
Acenaphthene	ACE	83-32-9	$C_{12}H_{10}$	154.21	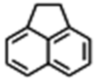	2.53	2.15E-3	1.84E-4	3.92	3.40	6.31	4.43/900/1.8E3/8.1E3	179
Fluorene	FLO	86-73-7	$C_{13}H_{10}$	166.22	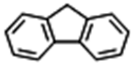	1.34	6.00E-4	9.62E-5	4.18	3.63	6.79	19.7/360/720/3.24E3	266
Phenanthrene	PHE	1985/1/8	$C_{14}H_{10}$	178.23	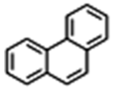	0.68	1.21E-4	4.23E-5	4.46	3.87	7.57	19.7/1.44E3/2.88E3/1.3E4	1865
Anthracene	ANT	120-12-7	$C_{14}H_{10}$	178.23	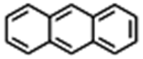	0.69	6.53E-6	5.56E-5	4.45	3.86	7.55	6.42/1.44E3/2.88E3/1.3E4	401
Fluoranthene	FLT	206-44-0	$C_{16}H_{10}$	202.25	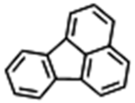	0.13	9.22E-6	8.86E-6	5.16	4.48	8.88	23.3/1.44E3/2.88E3/1.3E4	1179
Pyrene	PYR	129-00-0	$C_{16}H_{10}$	202.25	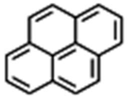	0.22	4.50E-6	1.19E-5	4.88	4.24	8.80	5.13/1.44E3/2.88E3/1.3E4	771

Chemicals	Abbreviation	CAS#	Formula	M.W.	Structure	Solubility (mg L^{-1}) 25 °C	Vapor pressure (mmHg) 25 °C	Henry's law constant (atm/m^3/mol) 25 °C	log K_{OW}	log K_{OC}	log K_{OA}	Half-life time (h) (Level III Fugacity Model; Air/Water/Soil/Sediment)	BCF (L kg^{-1})
Benzo[a]anthracene	BaA	56-55-3	$C_{18}H_{12}$	228.29	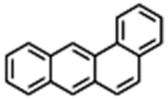	0.03	2.10E-7	1.20E-5	5.76	5.00	9.07	5.13/1.44E3/2.88E3/1.3E4	2934
Chrysene	CHR	218-01-9	$C_{18}H_{12}$	228.29	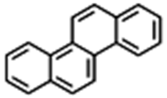	0.03	6.23E-9	5.23E-6	5.81	5.04	9.48	5.13/1.44E3/2.88E3/1.3E4	3165
Benzo[b]fluoranthene	BbF	205-99-2	$C_{20}H_{12}$	252.31	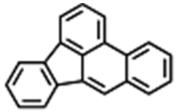	0.02	5.00E-7	6.57E-7	5.78	5.02	10.4	13.8/1.44E3/2.88E3/1.3E4	3024
Benzo[k]fluoranthene	BkF	207-08-9	$C_{20}H_{12}$	252.31	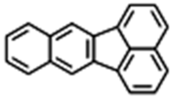	0.01	9.65E-10	5.84E-7	6.11	5.30	10.7	4.79/1.44E3/2.88E3/1.3E4	4993
Benzo[a]pyrene	BaP	50-32-8	$C_{20}H_{12}$	252.31	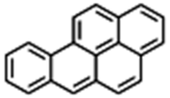	0.01	5.49E-9	4.57E-7	6.13	5.32	10.9	5.13/1.44E3/2.88E3/1.3E4	5147
Ideno-[1,2,3-c,d]pyrene	IcdP	193-39-5	$C_{22}H_{12}$	276.33	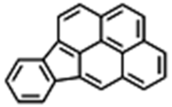	2.00E-3	1.25E-10	3.48E-7	6.70	5.82	11.6	3.98/1.44E3/2.88E3/1.3E4	1.22E4
Dibenzo[a,h]anthracene	DahA	53-70-3	$C_{22}H_{14}$	278.35	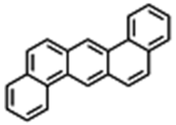	3.00E-3	9.55E-10	1.41E-7	6.75	5.68	11.8	5.13/1.44E3/2.88E3/1.3E4	9596

Chemicals	Abbreviation	CAS#	Formula	M.W.	Structure	Solubility (mg L^{-1}) 25 °C	Vapor pressure (mmHg) 25 °C	Henry's law constant (atm/m^3/mol) 25 °C	log K_{OW}	log K_{OC}	log K_{OA}	Half-life time (h) (Level III Fugacity Model; Air/Water/Soil/Sediment)	BCF (L kg^{-1})
Benzo[g,i,h]perylene	BghiP	191-24-2	$C_{22}H_{12}$	276.33	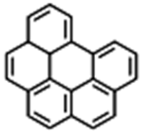	3.00E-3	1.00E-10	3.31E-7	6.63	5.75	11.5	2.95/1.44E3/2.88E3/1.3E4	1.10E4
					**O-PAH**								
Naphthalene-1-aldehyde	NAP-1-ALD	66-77-3	$C_{11}H_8O$	156.18	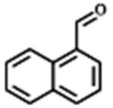	244.20	2.50E-3	1.94E-6	2.89	2.29	7.16	9.22/360/720/3.24E3	37
9-Fluorenone	9-FLO	486-25-9	$C_{13}H_8O$	180.2	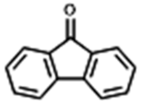	3.74	5.72E-5	6.77E-7	3.58	3.10	8.14	41.6/360/720/3.24E3	28
Anthraquinone	ATQ	84-65-1	$C_{14}H_8O_2$	208.21	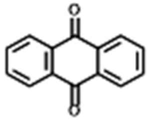	3.92	1.16E-7	3.18E-9	3.39	3.70	9.41	171/720/1.8E3/8.1E3	21
Phenanthrene-9-aldehyde	PHE-9-ALD	4707-71-5	$C_{15}H_{10}O$	206.24	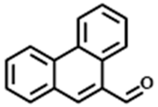	1.07	5.74E-6	1.34E-7	4.06	2.94	9.34	10.9/360/720/3.24E3	1019
Benzanthrone	BZO	82-05-3	$C_{17}H_{10}O$	230.26	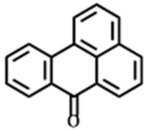	0.18	2.21E-7	6.61E-8	4.81	3.78	10.38	14.3/900/1.8E3/8.1E3	180
					**N-PAH**								

Chemicals	Abbreviation	CAS#	Formula	M.W.	Structure	Solubility (mg L^{-1}) 25 °C	Vapor pressure (mmHg) 25 °C	Henry's law constant (atm/m^3/mol) 25 °C	log K_{OW}	log K_{OC}	log K_{OA}	Half-life time (h) (Level III Fugacity Model; Air/Water/Soil/Sediment)	BCF (L kg^{-1})
1-Nitronaphthalene	1N-NAP	86-57-7	$C_{10}H_7NO_2$	173.17	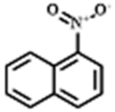	45.66	4.80E-4	1.76E-6	3.19	2.91	7.33	47.5/900/1.8E3/8.1E3	59
2-Nitronaphthalene	2N-NAP	581-89-5	$C_{10}H_7NO_2$	173.17	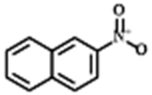	41.38	2.83E-4	3.08E-6	3.24	2.94	7.31	45.8/900/1.8E3/8.1E3	64
5-Nitroacenaphthene	5N-ACE	602-87-9	$C_{12}H_9NO_2$	199.20	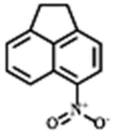	0.72	3.85E-5	4.88E-7	3.85	3.27	8.19	30.9/900/1.8E3/8.1E3	161
2-Nitrofluorene	2N-FLU	607-57-8	$C_{13}H_9NO_2$	211.22	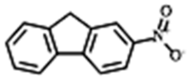	1.60	4.43E-6	2.87E-7	3.37	3.01	7.94	61.7/900/1.8E3/8.1E3	78
9-Nitroanthracene	9N-ANT	602-60-8	$C_{14}H_9NO_2$	223.23	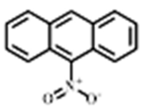	0.09	1.2E-6	2.13E-7	4.78	3.79	9.86	51.4/900/1.8E3/8.1E3	662
3-Nitrofluoranthene	3N-FLT	892-21-7	$C_{16}H_9NO_2$	247.25	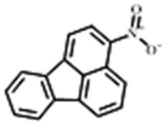	0.07	5.52E-8	2.45E-8	4.75	3.77	10.62	52/4.32E3/8.64E3/3.89E4	633
1-Nitropyrene	1N-PYR	5522-43-0	$C_{16}H_9NO_2$	247.25	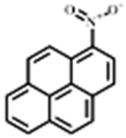	0.04	8.29E-8	2.45E-8	5.06	3.94	10.93	41.1/4.32E3/8.64E3/3.89E4	1013

Chemicals	Abbreviation	CAS#	Formula	M.W.	Structure	Solubility (mg L-1) 25 °C	Vapor pressure (mmHg) 25 °C	Henry's law constant (atm/m3/mol) 25 °C	log K_{OW}	log K_{OC}	log K_{OA}	Half-life time (h) (Level III Fugacity Model; Air/Water/Soil/Sediment)	BCF (L kg-1)
7-Nitrobenz[A]anthracene	7N-BaA	20268-51-3	$C_{18}H_{11}NO_2$	273.30	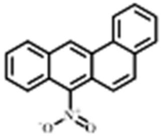	0.02	7.61E-9	1.47E-8	5.34	4.10	11.43	41.1/4.32E3/8.64E3/3.89E4	1547
6-Nitrochrysene	6N-CHR	7496-02-8	$C_{18}H_{11}NO_2$	273.30	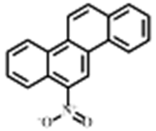	0.02	3.52E-9	1.47E-8	5.34	4.10	11.43	41.1/4.32E3/8.64E3/3.89E4	1547
						**Br-PAH**							
5-Bromoacenaphthene	5-BrACE	2051-98-1	$C_{12}H_9Br$	233.10	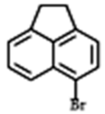	0.11	5.00E-9	1.13E-4	5.04	4.37	7.38	8.78/900/1.8E3/8.1E3	984
1-Bromoanthracene	1-BrANT	7397-92-4	$C_{14}H_9Br$	257.13	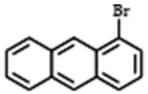	0.06	1.06E-5	2.05E-5	5.24	4.55	8.32	10.2/900/1.8E3/8.1E3	1321
9-Bromophenanthrene	9-BrPHE	573-17-1	$C_{14}H_9Br$	257.13	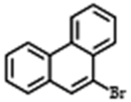	0.06	3.44E-5	2.05E-5	5.24	4.55	8.32	31.4/900/1.8E3/8.1E3	6053
3-Bromophenanthrene	3-BrPHE	715-50-4	$C_{14}H_9Br$	257.13	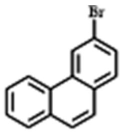	0.06	1.06E-5	2.05E-5	5.24	4.55	8.32	31.4/900/1.8E3/8.1E3	6053

Chemicals	Abbreviation	CAS#	Formula	M.W.	Structure	Solubility (mg L^{-1}) 25 °C	Vapor pressure (mmHg) 25 °C	Henry's law constant (atm/m^3/mol) 25 °C	log K_{OW}	log K_{OC}	log K_{OA}	Half-life time (h) (Level III Fugacity Model; Air/Water/Soil/Sediment)	BCF (L kg^{-1})
2-Bromophenanthrene	2-BrPHE	62162-97-4	$C_{14}H_9Br$	257.13	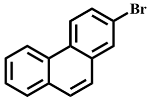	0.06	1.06E-5	2.05E-5	5.24	4.55	8.32	31.4/900/1.8E3/8.1E3	6053
2,7-Dibromofluorene	2,7-Br_2FLU	16433-88-8	$C_{13}H_8Br_2$	324.01	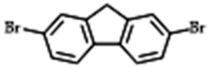	8.00E-03	7.04E-6	2.66E-5	5.80	5.03	8.76	57.8/1.44E3/2.88E3/1.3E4	3096
3-Bromofluoranthene	3-BrFLT	13438-50-1	$C_{16}H_9Br$	281.15	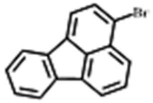	0.01	4.13E-7	3.31E-6	5.82	5.05	9.69	22.7/4.32E3/6.64E3/3.89E4	3229
1,8-Dibromoanthracene	1,8-Br_2ANT	131276-24-9	$C_{14}H_8Br_2$	336.02	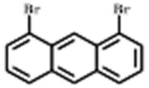	3.00E-3	9.89E-7	8.15E-6	6.13	5.32	9.61	35.6/1.44E3/2.88E3/1.3E4	1.97E4
1-Bromopyrene	1-BrPYR	1714-29-0	$C_{16}H_9Br$	281.15	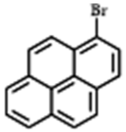	0.01	4.13E-7	3.31E-6	5.81	5.04	9.68	8.16/4.32E3/8.64E3/3.89E4	3165
2-Bromotriphenylene	2-BrTriPH	19111-87-6	$C_{18}H_{11}Br$	307.19	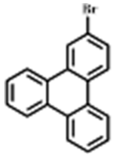	3.00E-3	5.84E-8	2.00E-6	6.41	5.56	10.5	8.16/4.32E3/8.64E3/3.89E4	7888
1,6-Dibromopyrene	1,6-Br_2PYR	27973-29-1	$C_{16}H_8Br_2$	360.04	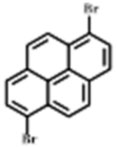	7.00E-4	3.95E-8	1.32E-6	6.69	5.84	11.0	28.5/4.32E3/8.64E3/3.89E4	4.94E4

Chemicals	Abbreviation	CAS#	Formula	M.W.	Structure	Solubility (mg L^{-1}) 25 °C	Vapor pressure (mmHg) 25 ˚C	Henry's law constant (atm/m^3/mol) 25 ˚C	log K_{OW}	log K_{OC}	log K_{OA}	Half-life time (h) (Level III Fugacity Model; Air/Water/Soil/Sediment)	BCF (L kg^{-1})
7-Bromo Benz[a]anthracene	7-BrBaA	32795-84-9	$C_{18}H_{11}Br$	307.19	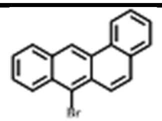	0.003	5.84E-8	2.00E-6	6.41	5.56	10.5	8.16/4.32E3/8.64E3/3.89E4	7888
6-Bromobenzo[a]pyrene	6-BrBaP	21248-00-0	$C_{20}H_{11}Br$	331.21	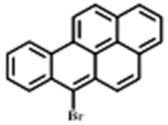	7.00E-4	2.18E-9	3.23E-7	7.00	6.08	11.9	8.16/4.32E3/8.64E3/3.89E4	1.93E4
**Cl-PAH**													
9-Chloroanthracene	9-ClANT	716-53-0	$C_{14}H_9Cl$	212.67	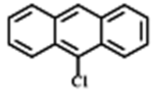	0.09	2.56E-5	3.80E-5	5.27	4.57	8.08	9.12/900/1.8E3/8.1E3	1394
9-Chlorophenanthrene	9-ClPHE	947-72-8	$C_{14}H_9Cl$	212.67	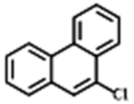	0.16	2.56E-5	3.80E-5	4.99	4.33	7.80	28.1/900/1.8E3/8.1E3	4168
2,7-Dichlorofluorene	2,7-Cl_2FLU	7012-16-0	$C_{13}H_8Cl_2$	235.11	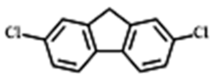	0.07	2.55E-5	9.19E-5	5.30	4.60	7.73	52.5/1.44E3/2.88E3/1.3E4	1469
9,10-Dichloro-phenanthrene	9,10-Cl_2PHE	17219-94-2	$C_{14}H_8Cl_2$	247.12	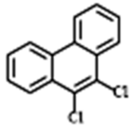	0.03	6.20E-6	2.82E-5	5.63	4.89	8.57	96.1/1.44E3/2.88E3/1.3E4	4.28E4
9,10-Dichloro-anthracene	9,10-Cl_2ANT	605-48-1	$C_{14}H_8Cl_2$	247.12	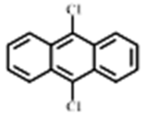	0.03	6.20E-6	2.82E-5	5.63	4.89	8.57	31.2/1.44E3/2.88E3/1.3E4	9339

Chemicals	Abbreviation	CAS#	Formula	M.W.	Structure	Solubility (mg L^{-1}) 25 °C	Vapor pressure (mmHg) 25 °C	Henry's law constant (atm/m^3/mol) 25 °C	log K_{OW}	log K_{OC}	log K_{OA}	Half-life time (h) (Level III Fugacity Model; Air/Water/Soil/Sediment)	BCF (L kg^{-1})
1-Chloropyrene	1-ClPYR	34244-14-9	$C_{16}H_9Cl$	236.69	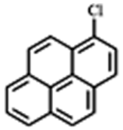	0.04	1.12E-6	6.15E-6	5.58	4.84	9.18	7.3/4.32E3/8.64E3/3.89E4	2223
1,5,9,10-Tetrachloro-anthracene	1,5,9,10-Cl_4ANT	82843-47-8	$C_{14}H_6Cl_4$	316.01	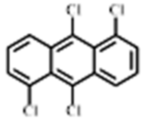	9.00E-4	3.43E-7	1.55E-5	6.92	4.90	10.1	152/4.32E3/8.64E3/3.89E4	6.62E4

Abbreviations: M.W.= Molecular Weight; CAS#= Chemical Abstracts Service; K_{OW}=Octanol-Water Partition Coefficient; K_{OC}=Soil Adsorption Coefficient; K_{OA}=Octanol-Air Partition Coefficient; BCF=Bioconcentration Factor;

The physicochemical properties of the pollutants were predicted using the USEPA EPI Suite software (EPIWEB 4.1)

Table S2. Basic information on internal standards used in the study.

NO.	Abbreviation	Chemicals	CAS#
1	NAP-d8	Naphthalene-d8	1146-65-2
2	ACY-d8	Acenaphthylene-d8	93951-97-4
3	ACE-d10	Acenaphthene-d10	15067-26-2
4	FLO-d10	Fluorene-d10	81103-79-9
5	PHE-d10	Phenanthrene-d10	1517-22-2
6	ANT-d10	Anthracene-d10	1719-06-8
7	FLT-d10	Fluoranthene-d10	93951-69-0
8	PYR-d10	Pyrene-d10	1718-52-1
9	BaA-d12	Benzo[a]anthracene-d12	1718-53-2
10	CHR-d12	Chrysene-d12	1719-03-5
11	BbF -d12	Benzo[b]fluoranthene-d12	93951-98-5
12	BkF-d12	Benzo[k]fluoranthene-d12	93952-01-3
13	BaP-d12	Benzo[a]pyrene-d12	93951-66-7
14	IcdP-d12	Ideno-[1,2,3-c,d]pyrene-d12	203578-33-0
15	DahA-d14	Dibenzo[a,h]anthracene-d14	13250-98-1
16	BghiP-d12	Benzo[g,i,h]perylene-d12	93951-66-7
17	1N-NAP-d7	1-Nitropyrene-d7	80789-77-1
18	9-FLO-d8	9-Fluorenone-d8	137219-34-2
19	ATQ-d8	Anthraquinone-d8	10439-39-1
20	9N-ANT-d9	9-Nitroanthracene-d9	220381-38-4
21	3N-FLT-d9	3-Nitrofluoranthene-d9	350820-11-0
22	1N-PYR-d9	1-Nitropyrene-d9	3487-20-8

Table S3. Sampling sites information

Sampling location	Longitude	Latitude
D-1	121.620498	38.957681
D-2	121.619314	38.956873
D-3	121.618996	38.957911
D-4	121.619596	38.958529
D-5	121.606076	38.952593
D-6	121.605515	38.953148
D-7	121.609095	38.951996
D-8	121.610179	38.952491
D-9	121.609629	38.966964
D-10	121.608333	38.965429

Table S4. Instrumental parameters of GC-HRMS

**GC conditions**				
GC system	Trace 1610 GC			
Sample wash volume	2.0 μL			
Sample injection volume	2.0 μL			
Inlet parameters	Splitless			
Column	TG- 5 MS column (30 m×0.25 mm,0.25 um)			
Aux Heaters (1 and 2)	280℃			
**GC oven settings**				
NO.	Retention time (min)	Rate (℃/min)	Target value (℃)	Hold time (min)
1	0.00		Run	
2	1.50	0.00	40.0	1.50
3	5.00	25.00	90.0	1.50
4	10.1	25.00	180	1.50
5	31.6	5.00	280	1.50
6	44.6	10.00	310	10.0
8		Stop run		
**MS conditions**				
MS system	Orbitrap Exploris 120 MS (Thermo Fisher Scientific)			
Global parameters	Ion source type	EI		
	Ion source temperature	250 ℃		
	Electron energy	70 eV		
	Emission lens	15 V		
	Emission current	50 μA		
Scan parameters (Full-scan)	Orbitrap resolution	3.0×10^{4}		
	Scan range (m/z)	50-750		
	AGC Target	Standard		
	Maximum Injection Time Mode	Auto		
	Microscans	1		
	C-Trap Offset	Static		

Table S5. Procedure blanks, matrix spiked recoveries, and method detection limits (MDLs) of target PAHs and derivatives

Target chemicals	Analyte m/z	Retention time (min)	Internal standard	Average procedure blank (n=8) (ng g^{-1})	Recovery (20 ng, n=8) [a]		MDLs (ng/g) [b]
					Average (%)	RSD	
NAP	128.06205	7.92	NAP-d8	5.61	91.3	0.003	2.29
ACY	152.06205	9.91	ACY -d8	n.d.	95.6	0.76	0.015
ACE	154.07770	10.21	ACE-d10	0.31	95.1	0.84	0.16
FLO	166.07770	11.16	FLO-d10	n.d.	96.3	2.26	0.018
PHE	178.07770	13.54	PHE-d10	n.d.	94.0	1.32	0.48
ANT	178.07770	13.67	ANT-d10	n.d.	95.5	0.66	0.49
FLT	202.07770	17.63	FLT-d10	n.d.	96.2	1.05	0.51
PYR	202.07770	18.47	PYR-d10	n.d.	95.3	2.66	0.53
BaA	228.09335	23.62	BaA -d12	4.72	92.0	0.64	0.053
CHR	228.09335	23.79	CHR-d12	n.d.	90.5	2.00	0.056
BbF	252.09335	28.20	BbF -d12	n.d.	96.6	0.14	0.14
BkF	252.09335	28.30	BkF-d12	n.d.	97.0	1.10	0.13
BaP	252.09335	29.43	BaP-d12	5.15	87.3	0.88	0.053
IcdP	276.09335	33.71	IcdP-d12	n.d.	92.2	4.88	0.57
DahA	278.10900	33.84	DahA-d14	n.d.	97.2	1.37	0.50
BghiP	276.09335	34.43	BghiP-d12	n.d.	98.6	2.37	0.52
NAP-1-ALD	156.05696	10.38	ACE-d10	0.84	95.0	0.77	0.068
9-FLO	180.05696	12.96	9-FLO-d8	n.d.	96.7	1.18	0.068
ATQ	208.05188	18.57	ATQ-d8	n.d.	72.3	1.40	0.13
PHE-9-ALD	206.07261	16.23	PYR-d10	6.54	99.2	1.32	0.10
BZO	230.07261	24.32	3N-FLT-d9	n.d.	93.7	3.31	0.39
1N-NAP	173.04713	11.39	1N-NAP-d7	5.88	89.7	0.92	0.093
2N-NAP	173.04713	11.84	1N-NAP-d7	n.d.	91.1	3.15	0.94
5N-ACE	199.06278	16.87	ATQ-d8	n.d.	76.2	1.42	1.17
2N-FLU	211.06278	18.37	PYR-d10	6.39	110	0.86	1.53
9N-ANT	223.06278	21.40	9N-ANT-d9	n.d.	114	0.41	3.49
3N-FLT	247.06278	25.14	3N-FLT-d9	n.d.	115	1.92	2.93
1N-PYR	247.06278	25.96	1N-PYR-d9	2.48	82.4	0.74	0.63
7N-BaA	273.07843	28.80	BbF -d12	8.09	97.5	1.33	1.04
6N-CHR	273.07843	30.02	BaP-d12	7.59	102	3.96	0.37
5-BrACE	231.98821	13.98	PHE-d10	1.46	94.5	1.73	0.045
1-BrANT	255.98821	18.08	PYR-d10	n.d.	95.6	2.12	0.19
9-BrPHE	255.98821	18.28	PYR-d10	n.d.	96.9	0.73	0.083
3-BrPHE	255.98821	18.28	PYR-d10	n.d.	97.0	2.30	0.20
2-BrPHE	255.98821	18.39	PYR-d10	n.d.	96.9	2.41	0.16
2,7-Br_2FLU	321.89872	20.61	9N-ANT-d9	n.d.	87.0	0.60	0.57
3-BrFLT	279.98821	22.80	BaA -d12	0.95	93.8	1.50	0.068
1,8-Br_2ANT	333.89872	23.31	BaA -d12	2.84	96.7	1.09	0.004
1-BrPYR	279.98821	23.76	CHR-d12	n.d.	101	3.67	0.43
2-BrTriPH	306.00386	28.58	BbF -d12	n.d.	94.0	1.07	0.56
1,6-Br_2PYR	357.89872	28.82	BkF-d12	n.d.	89.7	1.35	1.12
7-BrBaA	306.00386	28.86	BkF-d12	1.19	86.7	1.17	0.002
6-BrBaP	330.00386	34.53	BghiP-d12	4.63	93.6	1.56	0.038
9-ClANT	212.03872	16.54	ATQ-d8	n.d.	72.0	0.51	0.059
9-ClPHE	212.03872	16.80	ATQ-d8	n.d.	84.7	0.23	0.057
2,7-Cl_2FLU	233.99975	17.04	FLT-d10	n.d.	98.0	2.82	0.22
9,10-Cl_2PHE	245.99975	19.85	9N-ANT-d9	n.d.	84.6	0.020	0.053
9,10-Cl_2ANT	245.99975	20.07	9N-ANT-d9	n.d.	81.7	3.23	0.063
1-ClPYR	236.03872	21.90	BaA -d12	n.d.	97.0	1.80	0.074
1,5,9,10-Cl_4ANT	313.92181	27.69	BbF -d12	n.d.	94.0	0.71	0.46

a: Matrix spiked recoveries were calculated by the analyte concentration ratio of pre-spiked to post-spiked samples (both after subtracted matrix blank)
b: For the chemicals without procedure blank, MDLs were calculated based on instrumental LOQ and samples volume, while for the chemicals with procedure blank, MDLs were calculated based on 3 times of procedure blank's standard deviation and samples volume. RSD: relative standard deviation.
n.d. stands for not detected.

Table S6. Detail parameters of *GC EI with Statistics*.

**Main parameter settings**		
Peak detection settings	Mass tolerance	5 ppm
	Spectral peak S/N threshold	3
	Peak S/N threshold	3
	Smoothing	9
	TIC threshold	10^6
	Ion overlap window	95%
	Include reference and exception peaks	Ture
Group compounds settings	RT tolerance	5 sec
	Dot product threshold	500
	Composition threshold	10%
Library search settings	Search libraries	mainlib<br>replib<br>gc-orbitrap contaminants library<br>gc-orbitrap other environmental
	SI/RSI threshold	600

Table S7. Evaluation of Matrix Effects (ME) in Non-Targeted Screening.

Abbreviation	Formula	M.W.	RT (min)	Peak areas (internal standard)	Peak areas (sample)	ME (%)
NAP-d8	C10D8	136.11226	7.9	2.68E+06	3.13E+06	117
ACY-d8	C12D8	160.11226	9.89	8.01E+06	6.68E+06	83.3
ACE-d10	C12D10	164.14046	10.15	2.73E+06	3.04E+06	111
FLO-d10	C13D10	176.14046	11.1	6.97E+06	5.53E+06	79.4
PHE-d10	C14D10	188.14046	13.62	1.02E+07	1.08E+07	106
ANT-d10	C14D10	188.14046	13.62	1.32E+07	1.19E+07	89.9
FLT-d10	C16D10	212.14046	17.56	1.68E+07	1.60E+07	95.2
PYR-d10	C16D10	212.14046	18.39	1.95E+07	1.66E+07	85.2
BaA-d12	C18D12	240.16867	23.53	1.14E+07	9.25E+06	81.5
CHR-d12	C18D12	240.16867	23.68	1.96E+07	2.29E+07	117
BbF -d12	C20D12	264.16867	28.13	4.86E+06	4.12E+06	84.7
BkF-d12	C20D12	264.16867	28.23	6.31E+06	5.43E+06	86.0
BaP-d12	C20D12	264.16867	29.36	3.86E+06	3.37E+06	87.4
IcdP-d12	C22D12	288.16867	33.65	1.42E+06	1.24E+06	87.1
DahA-d14	C22D14	292.19687	33.76	1.29E+06	1.17E+06	90.8
BghiP-d12	C22D12	288.16867	34.37	1.32E+06	9.89E+05	74.8
1N-NAP-d7	C10D7NO2	180.09106	11.36	4.82E+05	4.89E+05	101
9-FLO-d8	C13D8O	188.10718	12.92	8.28E+06	6.58E+06	79.4
ATQ-d8	C14D8O2	216.10209	16.16	1.38E+06	1.38E+06	100
9N-ANT-d9	C14D9NO2	232.11927	18.81	4.59E+05	5.41E+05	118
3N-FLT-d9	C16D9NO2	232.11927	18.81	4.79E+05	5.41E+05	113
1N-PYR-d9	C16D9NO2	256.11927	25.93	9.10E+03	1.08E+04	119

Matrix effects were assessed by comparing the peak areas of internal standards in sample matrices to those in solvent standards.

Table S8. Literature reference values for TEFs used in TEQs calculation.

**Target chemicals**	**TEFs**	**References**
NAP	0.001	[1]
ACY	0.001	
ACE	0.001	
FLO	0.001	
PHE	0.001	
ANT	0.01	
FLT	0.001	
PYR	0.001	
BaA	0.1	
CHR	0.01	
BbF	0.1	
BkF	0.1	
BaP	1.0	
IcdP	0.1	
DahA	5.0	[2]
BghiP	0.01	[1]
9-FLO	0.002*	[3]
BZO	0.0039*	[3]
5N-ACE	0.01	[4]
2N-FLU	0.01	
9N-ANT	0.0032	
3N-FLT	0.0026	
1N-PYR	0.1	
6N-CHR	10	[4]
9-BrPHE	0.02*	[3]
1-BrPYR	0.04*	[3]
7-BrBaA	0.84*	[3]
9-ClPHE	0.03*	[3]
9,10-Cl_2ANT	0.2*	[3]

TEFs: toxic equivalency factor; *: relative potencies to benzo[a]pyrene (BaP); TEQs: toxic equivalence quantities.

Table S9. Parameters and references required for calculation of incremental lifetime cancer risk (ILCR).

Parameters	Abbreviation	Unit	Adults reference value	Children reference value	References
Body weight	BW	kg	70	15	[5]
Average life	AT	day	23433		[6]
Exposure frequency	EF	day $year^{-1}$	180		[7]
Exposure duration	ED	year	24	6	[8]
Ingestion rate	$IR_{Ingestion}$	mg day^{-1}	100	200	[8]
Inhalation rate	$IR_{Inhalation}$	m^3 day^{-1}	20	10	[9,10]
Skin surface area exposed	SA	cm^2	5700	2800	[8]
Adherence factor of dust to skin	AF	mg cm^{-2}	0.2	0.07	[8]
Dermal absorption factor	ABS	unitless	0.13		[8]
Particle emission factor	PEF	m^3 kg^{-1}	1.36×10^9		[11]

Table S10. Concentrations and detection frequency (DF) of target PAHs and their derivatives in dust samples.

	D-1	D-2	D-3	D-4	D-5	D-6	D-7	D-8	D-9	D-10	Min	Max	Median	DF (%)
**PAH**														
NAP	251	182	6.60	261	15.7	82.4	131	248	34.6	149	6.60	261	140	100
ACY	n.d.	n.d.	2.35	n.d.	0.688	n.d.	n.d.	n.d.	12.1	n.d.	0	12.1	n.d.	30
ACE	73.6	37.7	0.846	32.6	2.56	24.5	32.4	29.6	17.1	19.6	0.846	73.6	27.1	100
FLO	19.1	37.8	39.8	18.1	19.0	7.64	18.4	30.5	29.5	10.0	7.64	39.8	19.0	100
PHE	54.4	47.0	15.0	84.8	10.8	106	183	152	181	15.1	10.8	183	69.6	100
ANT	12.9	11.4	1.46	15.4	1.01	18.7	27.9	22.9	29.5	7.58	1.01	29.5	14.2	100
FLT	51.5	n.d.	16.3	57.7	n.d.	121	n.d.	119	142	4.30	0	142	33.9	70
PYR	44.8	5.71	14.5	62.6	6.09	124	124	129	223	10.9	5.71	223	53.7	100
BaA	12.8	2.85	0.160	16.7	n.d.	23.2	0.508	17.9	15.1	2.56	0	23.2	7.83	90
CHR	44.3	n.d.	10.2	38.0	3.12	58.4	69.7	48.1	160	3.82	0	160	41.2	90
BbF	37.0	n.d.	5.62	n.d.	3.38	57.3	68.7	59.9	n.d.	4.31	0	68.7	4.96	70
BkF	17.5	8.41	8.48	12	3.87	26.3	29.5	18.5	14.5	9.15	3.87	29.5	13.1	100
BaP	15.5	5.74	4.72	151	2.56	31.2	31.2	26.9	4.10	5.11	2.56	151	10.6	100
IcdP	35.6	18.1	n.d.	30.7	2.64	46.5	52.8	45.9	21.5	21.9	0	52.8	26.3	90
DahA	n.d.	n.d.	n.d.	n.d.	n.d.	n.d.	n.d.	n.d.	n.d.	n.d.	n.d.	n.d.	n.d.	0
BghiP	7.79	5.84	n.d.	29.3	3.85	36.2	45.1	43.5	5.63	10.0	0	45.1	8.90	90
∑PPAHs	678	362	126	810	75.3	764	814	991	889	273				
**O-PAH**														
NAP-1-ALD	1.59	n.d.	n.d.	1.77	1.83	1.91	10.7	0.115	6.00	n.d.	0	10.7	1.68	70
9-FLO	29.5	19.7	2.74	30.2	1.71	33.2	68.1	66.3	28.3	11.1	1.71	68.1	28.9	100
ATQ	35.7	n.d.	18.1	21.8	1.99	n.d.	n.d.	51.1	95.6	n.d.	0	95.6	10.0	60
PHE-9-ALD	0.867	0.734	n.d.	2.67	n.d.	0.449	2.11	1.50	3.02	0.221	0	3.02	0.800	80
BZO	4.60	n.d.	46.2	n.d.	3.7	61.2	25.7	23.1	n.d.	n.d.	0	61.2	4.2	60
∑O-PAHs	72.2	20.4	67.1	56.5	9.27	96.8	107	142	133	11.4				
**N-PAH**														
1N-NAP	8.28	28.4	n.d.	3.7	n.d.	15.1	16.7	9.0	11.8	1.6	0	28.4	8.7	80
2N-NAP	20.4	20.1	3.28	18.5	n.d.	16.0	14.6	14.9	26.6	15.6	0	26.6	15.8	90

	D-1	D-2	D-3	D-4	D-5	D-6	D-7	D-8	D-9	D-10	Min	Max	Median	DF (%)
5N-ACE	66.0	102	n.d.	55.7	1.68	n.d.	67.7	63.7	129	n.d.	0	129	59.7	70
2N-FLU	36	27.4	n.d.	95.1	n.d.	14.8	5.09	14.7	78.2	26.4	0	95.1	20.6	80
9N-ANT	n.d.	n.d.	n.d.	n.d.	n.d.	n.d.	n.d.	n.d.	n.d.	n.d.	n.d.	n.d.	n.d.	0
3N-FLT	6.71	7.29	n.d.	n.d.	n.d.	19.1	7.17	7.76	6.28	4.18	0	19.1	6.50	70
1N-PYR	13.5	41.7	0.96	7.63	n.d.	11.8	2.00	15.9	9.00	6.99	0	41.7	8.31	90
7N-BaA	n.d.	n.d.	n.d.	n.d.	n.d.	n.d.	n.d.	n.d.	n.d.	n.d.	n.d.	n.d.	n.d.	0
6N-CHR	n.d.	n.d.	n.d.	9.21	n.d.	n.d.	5.01	n.d.	3.79	2.60	0	9.21	0	40
∑N-PAHs	151	227	4.24	190	1.68	76.8	118	126	265	57.3				
							**Br-PAH**							
5-BrACE	17.6	31.2	n.d.	19.3	n.d.	2.93	4.51	4.03	5.32	2.33	0	31.2	4.27	80
1-BrANT	n.d.	n.d.	n.d.	n.d.	n.d.	n.d.	n.d.	n.d.	n.d.	n.d.	n.d.	n.d.	n.d.	
9-BrPHE	n.d.	n.d.	n.d.	n.d.	n.d.	n.d.	n.d.	n.d.	n.d.	n.d.	n.d.	n.d.	n.d.	
3-BrPHE	n.d.	n.d.	n.d.	n.d.	n.d.	n.d.	n.d.	n.d.	n.d.	n.d.	n.d.	n.d.	n.d.	
2-BrPHE	n.d.	n.d.	n.d.	n.d.	n.d.	n.d.	n.d.	n.d.	n.d.	n.d.	n.d.	n.d.	n.d.	
2,7-Br_2FLU	n.d.	n.d.	n.d.	n.d.	n.d.	n.d.	n.d.	n.d.	n.d.	n.d.	n.d.	n.d.	n.d.	
3-BrFLT	11.1	5.97	n.d.	4.93	n.d.	12.4	n.d.	36.2	54.0	42.4	0	54.0	8.54	70
1,8-Br_2ANT	0.501	0.135	0.391	0.298	0.094	0.206	n.d.	0.013	n.d.	0.198	0	0.501	0.167	80
1-BrPYR	n.d.	n.d.	n.d.	n.d.	n.d.	n.d.	n.d.	n.d.	n.d.	n.d.	n.d.	n.d.	n.d.	
2-BrTriPH	n.d.	n.d.	n.d.	n.d.	n.d.	n.d.	n.d.	n.d.	n.d.	n.d.	n.d.	n.d.	n.d.	
1,6-Br_2PYR	17.3	14.7	n.d.	30	n.d.	14.7	16.9	10.0	24.6	9.9	0	29.8	14.7	80
7-BrBaA	0.732	1.06	16.3	n.d.	11.3	19.0	20.4	13.5	4.38	1.65	0	20.4	7.82	90
6-BrBaP	4.18	3.60	32.8	12.3	3.87	3.07	1.81	2.51	12.1	n.d.	0	32.8	3.73	90
∑Br-PAHs	51.4	56.7	49.5	66.7	15.2	52.4	43.7	66.3	100	56.5				
							**Cl-PAH**							
9-ClANT	n.d.	n.d.	0.656	n.d.	n.d.	n.d.	n.d.	n.d.	5.523	n.d.	0	5.52	n.d.	20
9-ClPHE	n.d.	n.d.	n.d.	n.d.	n.d.	n.d.	n.d.	8.03	9.261	n.d.	0	9.26	n.d.	20
2,7-Cl_2FLU	n.d.	n.d.	n.d.	n.d.	n.d.	n.d.	n.d.	n.d.	n.d.	n.d.	0	0	n.d.	0
9,10-Cl_2PHE	n.d.	n.d.	0.265	n.d.	n.d.	n.d.	n.d.	n.d.	n.d.	n.d.	0	0.265	n.d.	10
9,10-Cl_2ANT	n.d.	n.d.	0.306	n.d.	n.d.	n.d.	n.d.	n.d.	n.d.	n.d.	0	0.306	n.d.	10
1-ClPYR	n.d.	n.d.	n.d.	n.d.	n.d.	n.d.	n.d.	n.d.	n.d.	n.d.	0	0	n.d.	0
1,5,9,10-Cl_4ANT	n.d.	n.d.	n.d.	n.d.	n.d.	n.d.	n.d.	n.d.	n.d.	n.d.	0	0	n.d.	0
∑Cl-PAHs			1.23					8.03	14.8					

n.d. stands for not detected.

Table S11. Twenty-nine PACs identified by GC-Orbitrap-HRMS non-target screening analysis

No.	Name	Abbreviation	CAS#	Formula	RT (min)	M.W.	m/z	Error （ppm）	Structure	△RI	SI	Level
						Emerging PAH						
1	4H-cyclopenta[def]phenanthrene	4H-CPPHE	203-64-5	C15 H10	15.48	190.07770	190.07854	4.39338	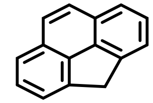	57	807	3
2	7H-benzanthrene△	7H-BEN		C17 H12	19.97	216.09335	216.09395	2.75993	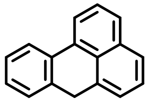		786	3
3	7H-benzo[c]fluorene	7H-BFLO	205-12-9	C17 H12	20.37	216.09335	216.09373	1.77137	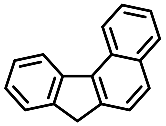	63	830	3
4	3,4-dihydro-cyclopenta(cd)pyrene△	3,4-DH-CP-PYR	25732-74-5	C18 H12	24.12	228.09335	228.09422	3.81887	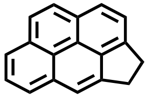		779	3
5	9H-cyclopenta[a]pyrene△	9H-CPPYR	50861-05-7	C19 H12	25.87	240.09335	240.09378	1.78496	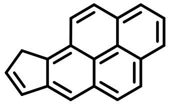		707	3
						Phenyl-PAH						
6	9-phenyl-9H-fluorene	9-Ph-9H-FLO	789-24-2	C19 H14	25.92	242.10900	242.10956	2.29683	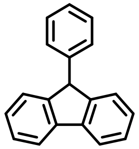		702	3
7	2-phenyl-phenanthrene△	2-Ph-PHE	4325-77-3	C20 H14	26.56	254.10900	254.10986	3.38933	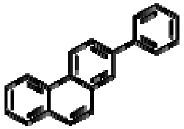		881	3

No.	Name	Abbreviation	CAS#	Formula	RT (min)	M.W.	m/z	Error （ppm）	Structure	△RI	SI	Level
					**O-PAH**							
8	Phenol	Phenol	108-95-2	C6 H6 O	5.87	94.04131	94.04146	1.50731	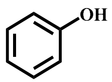	1	952	2b
9	7H-benz[de]anthracen-7-one	7H-BANT-7-O	82-05-3	C17 H10 O	23.05	230.07261	230.07339	3.38273	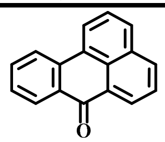	95	880	3
					**Alkyl-PAH**							
10	1,3-diethyl-5-methyl-benzene△	1,3-DE-5-MB	2050-24-0	C11 H16	7.52	148.12465	148.12503	2.55485	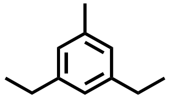	3	803	2a
11	1,2,3,4-tetramethyl-naphthalene	1,2,3,4-TM-NAP	3031-15-0	C14 H16	12.88	184.12465	184.12518	2.88405	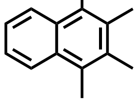	8	805	2b
12	9,9-dimethyl-9H-fluorene	9,9-DM-9H-FLO	4569-45-3	C15 H14	13.94	194.10900	194.10965	3.33646	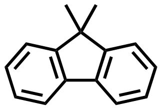		823	3
13	2,5-dimethyl-phenanthrene	2,5-DM-PHE	3674-66-6	C16 H14	17.38	206.10900	206.10939	1.88365	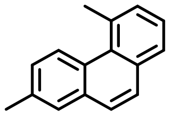		884	3
14	7-methyl-benz[a]anthracene	7-M-BANT	2541-69-7	C19 H14	25.72	242.10900	242.10971	2.92708	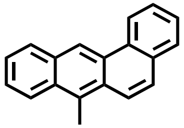		683	3
15	12-methyl-benz[a]anthracene△	12-M-BANT	2422-79-9	C19 H14	26.11	242.10900	242.10950	2.04474	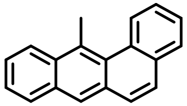		652	3
					**PAO/SH**							

No.	Name	Abbreviation	CAS#	Formula	RT (min)	M.W.	m/z	Error （ppm）	Structure	△RI	SI	Level
16	3,7-dimethyldibenzothiophene△	DMDBT	1136-85-2	C14 H12 S	15.99	212.06542	212.06613	3.34298	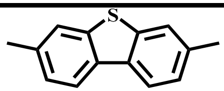		705	3
17	4b,10a-dihydro-benzo[b]benzo[3,4]cyclobuta[1,2-e][1,4]dioxin☆	DBBCD	42896-18-4	C14 H10 O2	17.85	210.06753	210.06810	2.70805	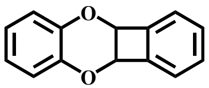		603	3
18	2H-phenanthro[9,10-b]pyran△	2H-PHEP	217-67-4	C17 H12 O	20.49	232.08826	232.08878	2.19345	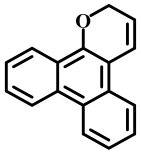		886	3
19	Benzo[b]naphtho[2,1-d]thiophene	BANPT	239-35-0	C16 H10 S	22.60	234.04977	234.05037	2.54929	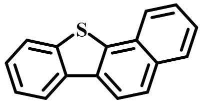	7	718	2b
20	1-phenyldibenzofuran	PhDBF		C18 H12 O	23.57	244.08826	244.08894	2.77326	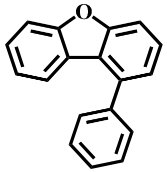		745	3
					**Others**							
21	Methyl salicylate△	Methyl salicylate	119-36-8	C8 H8 O3	7.96	152.04679	152.04718	2.52906	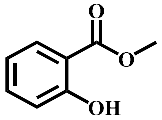	12	836	2b
22	Methyl-3-phenylpropionate△	M-PhPP	103-25-3	C10 H12 O2	8.51	164.08318	164.08360	2.57031	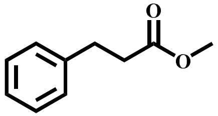	4	854	2a
23	1,1'-[1,2-ethanediylbis(oxy)]bis-benzene	EDB(O)BB		C14 H14 O2	13.64	214.09883	214.09920	1.71027	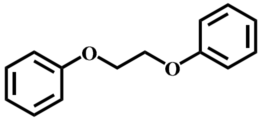		725	3

No.	Name	Abbreviation	CAS#	Formula	RT (min)	M.W.	m/z	Error （ppm）	Structure	△RI	SI	Level
24	2,4-diphenyl-4-methyl-2(E)-pentene△	DPhMP	22768-22-5	C18 H20	14.10	236.15595	236.15660	2.74713	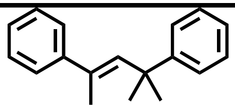	15	848	2a
25	5H-dibenzo[a,d]cycloheptene☆	5H-DBCH	256-81-5	C15 H12	15.11	192.09335	192.09396	3.18419	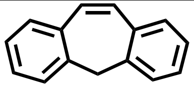		898	3
26	N-phenyl-2-naphthalenamine	N-Ph-NAPA	135-88-6	C16 H13 N	20.23	219.10425	219.10466	1.86689	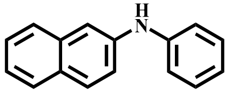	31	837	3
27	2,4-bis(1-methyl-1-phenylethyl)-phenol	BMPhEPh	2772-45-4	C24 H26 O	24.36	330.19781	330.19891	3.32101	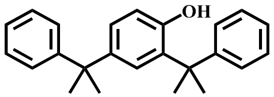	12	864	2a
28	9H-tribenzo[a,c,e]cycloheptene☆	9H-TBCH	213-10-5	C19 H14	25.57	242.10900	242.10954	2.23381	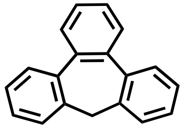		915	3
29	2-(2H-benzotriazol-2-yl)-4,6-bis(1,1-dimethylpropyl)-phenol	BTBDMPPh		C22 H29 N3 O	26.55	351.23051	351.23135	2.39070	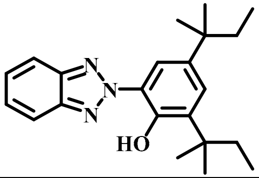		721	3

Abbreviations: M.W.= Molecular Weight; Phenyl-PAH= Phenyl PAH; O-PAH= Oxidized PAH; Alkyl-PAH= Alkylated PAH; PAOH= Oxygen/Sulfur-containing heterocyclic PAH; Others= Other aromatic contaminants; △RI= Retention index delta between base compound and library hit; SI= Library Search Index; level= Confidence level；

The calculation and definition methods for RI and level refer to Text S3;

△: first report in dust environment;

☆: first report in environment matrix.

Table S12. The LC_{50} (mg/L) of target and screening abbreviations for soil organisms (earthworm) and aquatic organisms (fish).

Abbreviation	SMILES	LC_{50} (mg/L)	
		**Earthworm (14d)**	**Fish (96h)**
NAP	C1=CC=C2C=CC=CC2=C1	169	9.39
ACY	C1=CC2=C3C(=C1)C=CC3=CC=C2	167	2.28
ACE	C1CC2=CC=CC3=C2C1=CC=C3	160	1.48
FLO	C1C2=CC=CC=C2C3=CC=CC=C31	179	2.11
PHE	C1=CC=C2C(=C1)C=CC3=CC=CC=C32	177	1.15
ANT	C1=CC=C2C=C3C=CC=CC3=CC2=C1	177	1.15
FLT	C1=CC=C2C(=C1)C3=CC=CC4=C3C2=CC=C4	175	0.386
PYR	C1=CC2=C3C(=C1)C=CC4=CC=CC(=C43)C=C2	175	0.386
BaA	C1=CC=C2C(=C1)C=CC3=CC4=CC=CC=C4C=C32	171	0.129
CHR	C1=CC=C2C(=C1)C=CC3=C2C=CC4=CC=CC=C43	171	0.129
BbF	C1=CC=C2C3=C4C(=CC=C3)C5=CC=CC=C5C4=CC2=C1	164	0.042
BkF	C1=CC=C2C=C3C4=CC=CC5=C4C(=CC=C5)C3=CC2=C1	164	0.042
BaP	C1=CC=C2C3=C4C(=CC2=C1)C=CC5=C4C(=CC=C5)C=C3	164	0.042
IcdP	C1=CC=C2C(=C1)C3=C4C2=CC5=CC=CC6=C5C4=C(C=C6)C=C3	157	0.014
DahA	C1=CC=C2C(=C1)C=CC3=CC4=C(C=CC5=CC=CC=C54)C=C32	158	0.014
BghiP	C1=CC2=C3C(=C1)C4=CC=CC5=C4C6=C(C=C5)C=CC(=C36)C=C2	157	0.014
NAP-1-ALD	C1=CC=C2C(=C1)C=CC=C2C=O	220	20.5
9-FLO	C1=CC=C2C(=C1)C3=CC=CC=C3C2=O	216	5.99
ATQ	C1=CC=C2C(=C1)C(=O)C3=CC=CC=C3C2=O	263	10.6
PHE-9-ALD	C1=CC=C2C(=C1)C=C(C3=CC=CC=C23)C=O	219	2.38
BZO	C1=CC=C2C(=C1)C3=CC=CC4=C3C(=CC=C4)C2=O	209	0.673
1N-NAP	C1=CC=C2C(=C1)C=CC=C2[N+](=O)[O-]	238	18.5

Abbreviation	SMILES	LC_{50} (mg/L)	
		Earthworm (14d)	Fish (96h)
2N-NAP	C1=CC=C2C=C(C=CC2=C1)[N+](=O)[O-]	238	18.5
5N-ACE	C1CC2=CC=C(C3=CC=CC1=C23)[N+](=O)[O-]	216	2.79
2N-FLU	C1C2=CC=CC=C2C3=C1C=C(C=C3)[N+](=O)[O-]	237	3.92
9N-ANT	C1=CC=C2C(=C1)C=C3C=CC=CC3=C2[N+](=O)[O-]	232	2.09
3N-FLT	O=N(=O)C2CCC3C1CCCCC1C4CCCC2C34	224	0.44
1N-PYR	C1=CC2=C3C(=C1)C=CC4=C(C=CC(=C43)C=C2)[N+](=O)[O-]	223	0.688
7N-BaA	C1=CC=C2C(=C1)C=CC3=C(C4=CC=CC=C4C=C32)[N+](=O)[O-]	214	0.225
6N-CHR	C1=CC=C2C(=C1)C=CC3=C2C=C(C4=CC=CC=C34)[N+](=O)[O-]	214	0.225
5-BrACE	C1CC2=CC=C(C3=CC=CC1=C23)Br	196	0.356
1-BrANT	C1=CC=C2C=C3C(=CC2=C1)C=CC=C3Br	206	0.263
9-BrPHE	C1=CC=C2C(=C1)C=C(C3=CC=CC=C23)Br	206	0.263
3-BrPHE	C1=CC=C2C(=C1)C=CC3=C2C=C(C=C3)Br	206	0.263
2-BrPHE	C1=CC=C2C(=C1)C=CC3=C2C=CC(=C3)Br	206	0.263
2,7-Br_2FLU	C1C2=C(C=CC(=C2)Br)C3=C1C=C(C=C3)Br	228	0.104
3-BrFLT	C1=CC=C2C(=C1)C3=C4C2=CC=CC4=C(C=C3)Br	196	0.085
1,8-Br_2ANT	C1=CC2=CC3=C(C=C2C(=C1)Br)C(=CC=C3)Br	218	0.054
1-BrPYR	C1=CC2=C3C(=C1)C=CC4=C(C=CC(=C43)C=C2)Br	196	0.085
2-BrTriPH	C1=CC=C2C(=C1)C3=C(C=C(C=C3)Br)C4=CC=CC=C24	186	0.028
1,6-Br_2PYR	C1=CC2=C(C=CC3=C2C4=C1C=CC(=C4C=C3)Br)Br	203	0.017
7-BrBaA	C1=CC=C2C(=C1)C=CC3=C(C4=CC=CC=C4C=C32)Br	186	0.028
6-BrBaP	C1=CC=C2C(=C1)C3=C4C(=C2Br)C=CC5=CC=CC(=C54)C=C3	175	0.0088
9-ClANT	C1=CC=C2C(=C1)C=C3C=CC=CC3=C2Cl	181	0.361
9-ClPHE	C1=CC=C2C(=C1)C=C(C3=CC=CC=C23)Cl	181	0.361
2,7-Cl_2FLU	C1C2=C(C=CC(=C2)Cl)C3=C1C=C(C=C3)Cl	186	0.208

	Abbreviation	SMILES	LC_{50} (mg/L)	
			Earthworm (14d)	Fish (96h)
	9,10-Cl_2PHE	C1=CC=C2C(=C1)C3=CC=CC=C3C(=C2Cl)Cl	180	0.111
	9,10-Cl_2ANT	C1=CC=C2C(=C1)C(=C3C=CC=CC3=C2Cl)Cl	180	0.111
	1-ClPYR	C1=CC2=C3C(=C1)C=CC4=C(C=CC(=C43)C=C2)Cl	175	0.119
	1,5,9,10-Cl_4ANT	C1=CC2=C(C(=C1)Cl)C(=C3C=CC=C(C3=C2Cl)Cl)Cl	170	0.0098
1	4H-CPPHE	C1C2=CC=CC3=C2C4=C(C=CC=C41)C=C3	178	0.717
2	7H-BEN	C1C2=CC=CC=C2C3=CC=CC4=C3C1=CC=C4	175	0.242
3	7H-BFLO	C1C2=C(C3=CC=CC=C31)C4=CC=CC=C4C=C2	175	0.242
4	3,4-DH-CP-PYR	C1CC2=CC3=CC=CC4=C3C5=C(C=CC1=C25)C=C4	156	0.057
5	9H-CPPYR	C1C=CC2=C1C3=C4C(=C2)C=CC5=C4C(=CC=C5)C=C3	154	0.034
6	9-Ph-9H-FLO	C1=CC=C(C=C1)C2C3=CC=CC=C3C4=CC=CC=C24	189	0.189
7	2-Ph-PHE	C1=CC=C(C=C1)C2=CC3=C(C=C2)C4=CC=CC=C4C=C3	166	0.043
8	Phenol	C1=CC=C(C=C1)O	184	212
9	7H-BANT-7-O	C1=CC=C2C(=C1)C3=CC=CC4=C3C(=CC=C4)C2=O	209	0.673
10	1,3-DE-5-MB	CCC1=CC(=CC(=C1)C)CC	138	0.543
11	1,2,3,4-TM-NAP	CC1=C(C2=CC=CC=C2C(=C1C)C)C	144	0.146
12	9,9-DM-9H-FLO	CC1(C2=CC=CC=C2C3=CC=CC=C31)C	181	0.733
13	2,5-DM-PHE	CC1(C2=CC=CC=C2C3=CC=CC=C31)C	181	0.138
14	7-M-BANT	CC1=C2C=CC3=CC=CC=C3C2=CC4=CC=CC=C14	159	0.044
15	12-M-BANT	CC1=C2C(=CC3=CC=CC=C13)C=CC4=CC=CC=C42	159	0.044
16	DMDBT	CC1=CC2=C(C=C1)C3=C(S2)C=C(C=C3)C	169	0.206
17	DBBCD	C1=CC=C2C3C(C2=C1)OC4=CC=CC=C4O3	274	14.3
18	2H-PHEP	C1C=CC2=C(O1)C3=CC=CC=C3C4=CC=CC=C24	191	0.291
19	BANPT	C1=CC=C2C(=C1)C=CC3=C2SC4=CC=CC=C34	183	0.192

	Abbreviation	SMILES	LC_{50} (mg/L)	
			Earthworm (14d)	Fish (96h)
20	PhDBF	C1=CC=C(C=C1)C2=C3C4=CC=CC=C4OC3=CC=C2	185	0.151
21	Methyl salicylate	COC(=O)C1=CC=CC=C1O	229	35.9
22	M-PhPP	COC(=O)CCC1=CC=CC=C1	249	41.3
23	EDB(O)BB	C1=CC=C(C=C1)OCCOC2=CC=CC=C2	242	4.20
24	DPhMP	C/C(=C/C(C)(C)C1=CC=CC=C1)/C2=CC=CC=C2	143	0.020
25	5H-DBCH	C1C2=CC=CC=C2C=CC3=CC=CC=C31	172	0.50
26	N-Ph-NAPA	C1=CC=C(C=C1)NC2=CC3=CC=CC=C3C=C2	212	1.10
27	BMPhEPh	CC(C)(C1=CC=CC=C1)C2=CC(=C(C=C2)O)C(C)(C)C3=CC=CC=C3	186	0.015
28	9H-TBCH	C12=CC=CC=C1C1=CC=CC=C1C1=CC=CC=C1C2	171	0.08
29	BTBDMPPh	CCC(C)(C)C1=CC(=C(C(=C1)N2N=C3C=CC=CC3=N2)O)C(C)(C)CC	174	0.0055

Table S13. TEQs for target contaminants.

Contaminant	D-1	D-2	D-3	D-4	D-5	D-6	D-7	D-8	D-9	D-10
NAP	2.51E-04	1.82E-04	6.60E-06	2.61E-04	1.57E-05	8.24E-05	1.31E-04	2.48E-04	3.46E-05	1.49E-04
ACY	0	0	2.35E-06	0	6.88E-07	0	0	0	1.21E-05	0
ACE	7.36E-05	3.77E-05	8.46E-07	3.26E-05	2.56E-06	2.45E-05	3.24E-05	2.96E-05	1.71E-05	1.96E-05
FLO	1.91E-05	3.78E-05	3.98E-05	1.81E-05	1.90E-05	7.64E-06	1.84E-05	3.05E-05	2.95E-05	9.98E-06
PHE	5.44E-05	4.70E-05	1.50E-05	8.48E-05	1.08E-05	1.06E-04	1.83E-04	1.52E-04	1.81E-04	1.51E-05
ANT	1.29E-04	1.14E-04	1.46E-05	1.54E-04	1.01E-05	1.87E-04	2.79E-04	2.29E-04	2.95E-04	7.58E-05
FLT	5.15E-05	0	1.63E-05	5.77E-05	0	1.21E-04	0	1.19E-04	1.42E-04	4.30E-06
PYR	4.48E-05	5.71E-06	1.45E-05	6.26E-05	6.09E-06	1.24E-04	1.24E-04	1.29E-04	2.23E-04	1.09E-05
BaA	1.28E-03	2.85E-04	1.60E-05	1.67E-03	0	2.32E-03	5.08E-05	1.79E-03	1.51E-03	2.56E-04
CHR	4.43E-04	0	1.02E-04	3.80E-04	3.12E-05	5.84E-04	6.97E-04	4.81E-04	1.60E-03	3.82E-05
BbF	3.70E-03	0	5.62E-04	0	3.38E-04	5.73E-03	6.87E-03	5.99E-03	0	4.31E-04
BkF	1.75E-03	8.41E-04	8.48E-04	1.17E-03	3.87E-04	2.63E-03	2.95E-03	1.85E-03	1.45E-03	9.15E-04
BaP	1.55E-02	5.74E-03	4.72E-03	1.51E-01	2.56E-03	3.12E-02	3.12E-02	2.69E-02	4.10E-03	5.11E-03
IcdP	3.56E-03	1.81E-03	0	3.07E-03	2.64E-04	4.65E-03	5.28E-03	4.59E-03	2.15E-03	2.19E-03
BghiP	7.79E-05	5.84E-05	0	2.93E-04	3.85E-05	3.62E-04	4.51E-04	4.35E-04	5.63E-05	1.00E-04
∑PAHs	2.69E-02	9.16E-03	6.35E-03	1.58E-01	3.69E-03	4.81E-02	4.83E-02	4.30E-02	1.18E-02	9.32E-03
NAP-1-ALD	0	0	0	0	0	0	0	0	0	0
9-FLO	5.90E-05	3.94E-05	5.47E-06	6.03E-05	3.42E-06	6.65E-05	1.36E-04	1.33E-04	5.66E-05	2.23E-05
ATQ	0	0	0	0	0	0	0	0	0	0
PHE-9-ALD	0	0	0	0	0	0	0	0	0	0
BZO	1.79361E-05	0	1.80E-04	0	1.46E-05	2.39E-04	1.00E-04	9.00861E-05	0	0
∑O-PAHs	7.68861E-05	3.94E-05	1.86E-04	6.03E-05	1.80E-05	3.05E-04	2.36E-04	2.23E-04	5.66E-05	2.23E-05
1N-NAP	0	0	0	0	0	0	0	0	0	0
2N-NAP	0	0	0	0	0	0	0	0	0	0
5N-ACE	6.60E-04	1.02E-03	0	5.57E-04	1.68E-05	0	6.77E-04	6.37E-04	1.29E-03	0
2N-FLU	3.61E-04	2.74E-04	0	9.51E-04	0	1.48E-04	5.09E-05	1.47E-04	7.82E-04	2.64E-04
3N-FLT	1.74408E-05	1.89462E-05	0	0	0	4.98E-05	1.86E-05	2.02E-05	1.63E-05	1.09E-05
1N-PYR	1.35E-03	4.17E-03	9.57E-05	7.63E-04	0	1.18E-03	2.00E-04	1.59E-03	9.00E-04	6.99E-04
6N-CHR	0	0	0	9.21E-02	0	0	5.01E-02	0	3.79E-02	2.60E-02
∑N-PAHs	2.39E-03	5.49E-03	9.57E-05	9.44E-02	1.68E-05	1.38E-03	5.10E-02	2.39E-03	4.09E-02	2.69E-02

Contaminant	D-1	D-2	D-3	D-4	D-5	D-6	D-7	D-8	D-9	D-10
5-BrACE	0	0	0	0	0	0	0	0	0	0
3-BrFLT	0	0	0	0	0	0	0	0	0	0
1,8-Br_2ANT	0	0	0	0	0	0	0	0	0	0
1,6-Br_2PYR	0	0	0	0	0	0	0	0	0	0
7-BrBaA	6.14E-04	8.91E-04	1.37E-02	0	9.46E-03	1.60E-02	1.72E-02	1.13E-02	3.68E-03	1.39E-03
6-BrBaP	0	0	0	0	0	0	0	0	0	0
∑Br-PAHs	6.14E-04	8.91E-04	1.37E-02	0	9.46E-03	1.60E-02	1.72E-02	1.13E-02	3.68E-03	1.39E-03
9-ClANT	0	0	0	0	0	0	0	0	0	0
9-ClPHE	0	0	0	0	0	0	0	2.41E-04	2.78E-04	0
9,10-Cl_2PHE	0	0	0	0	0	0	0	0	0	0
9,10-Cl_2ANT	0	0	6.12E-05	0	0	0	0	0	0	0
∑Cl-PAHs	0	0	6.12E-05	0	0	0	0	2.41E-04	2.78E-04	0

Table S14. The results of ILCR analysis.

		CS	Adults				Children			
			$ILCR_{Ingestion}$	$ILCR_{Inhalation}$	$ILCR_{Dermal}$	ILCRs	$ILCR_{Ingestion}$	$ILCR_{Inhalation}$	$ILCR_{Dermal}$	ILCRs
∑PAHs	MIN	3.69E-03	7.09E-09	5.50E-13	3.60E-08	4.30E-08	9.89E-09	1.92E-13	4.32E-09	1.42E-08
	MAX	1.58E-01	3.05E-07	2.36E-11	1.55E-06	1.85E-06	4.25E-07	8.24E-12	1.86E-07	6.11E-07
	Mean	3.65E-02	7.02E-08	5.44E-12	3.56E-07	4.26E-07	9.80E-08	1.90E-12	4.27E-08	1.41E-07
	Median	1.93E-02	3.72E-08	2.88E-12	1.89E-07	2.26E-07	5.19E-08	1.01E-12	2.27E-08	7.46E-08
∑O-PAHs	MIN	1.80E-05	3.46E-11	2.69E-15	1.76E-10	2.10E-10	4.83E-11	9.37E-16	2.11E-11	6.94E-11
	MAX	3.05E-04	5.87E-10	4.55E-14	2.98E-09	3.56E-09	8.19E-10	1.59E-14	3.57E-10	1.18E-09
	Mean	1.22E-04	2.35E-10	1.82E-14	1.19E-09	1.43E-09	3.28E-10	6.37E-15	1.43E-10	4.72E-10
	Median	6.86E-05	1.32E-10	1.02E-14	6.69E-10	8.01E-10	1.84E-10	3.57E-15	8.04E-11	2.65E-10
∑N-PAHs	MIN	1.68E-05	3.23E-11	2.51E-15	1.64E-10	1.96E-10	4.52E-11	8.75E-16	1.97E-11	6.49E-11
	MAX	9.44E-02	1.81E-07	1.41E-11	9.21E-07	1.10E-06	2.53E-07	4.91E-12	1.11E-07	3.64E-07
	Mean	2.25E-02	4.32E-08	3.35E-12	2.20E-07	2.63E-07	6.04E-08	1.17E-12	2.63E-08	8.67E-08
	Median	3.94E-03	7.57E-09	5.87E-13	3.84E-08	4.60E-08	1.06E-08	2.05E-13	4.61E-09	1.52E-08
∑Br-PAHs	MIN	0	0	0	0	0	0	0	0	0
	MAX	1.72E-02	3.30E-08	2.56E-12	1.67E-07	2.00E-07	4.61E-08	8.93E-13	2.01E-08	6.62E-08
	Mean	7.42E-03	1.43E-08	1.11E-12	7.24E-08	8.66E-08	1.99E-08	3.86E-13	8.69E-09	2.86E-08
	Median	6.57E-03	1.26E-08	9.79E-13	6.41E-08	7.67E-08	1.76E-08	3.42E-13	7.69E-09	2.53E-08
∑Cl-PAHs	MIN	0	0	0	0	0	0	0	0	0
	MAX	2.78E-04	5.34E-10	4.14E-14	2.71E-09	3.25E-09	7.46E-10	1.45E-14	3.25E-10	1.07E-09
	Mean	5.80E-05	1.11E-10	8.65E-15	5.66E-10	6.77E-10	1.56E-10	3.02E-15	6.79E-11	2.24E-10
	Median	0	0	0	0	0	0	0	0	0
∑All	MIN	1.32E-02	2.53E-08	1.97E-12	1.29E-07	1.54E-07	3.54E-08	6.86E-13	1.54E-08	5.08E-08
	MAX	2.53E-01	4.86E-07	3.77E-11	2.47E-06	2.95E-06	6.79E-07	1.32E-11	2.96E-07	9.75E-07
	Mean	6.66E-02	1.28E-07	9.93E-12	6.50E-07	7.78E-07	1.79E-07	3.47E-12	7.80E-08	2.57E-07
	Median	4.72E-02	9.07E-08	7.03E-12	4.60E-07	5.51E-07	1.27E-07	2.45E-12	5.52E-08	1.82E-07

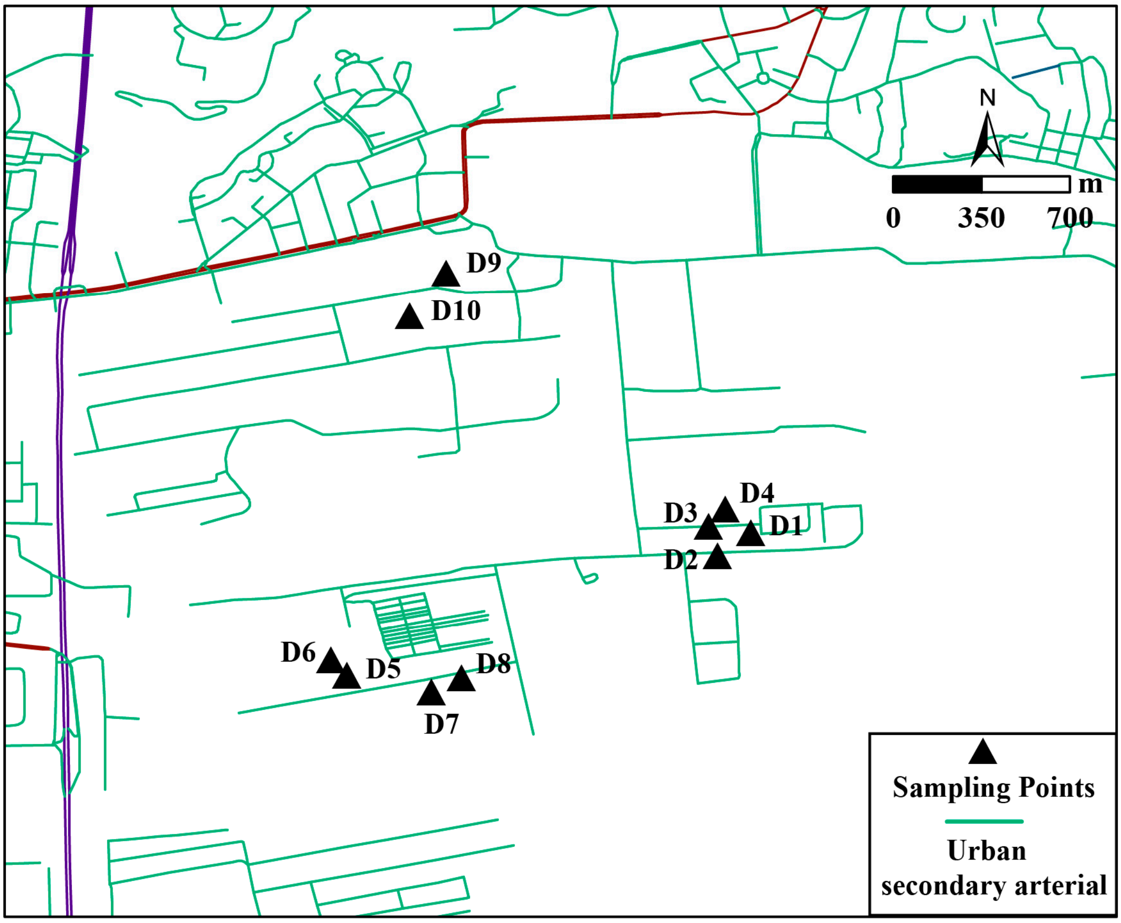

Figure S1. The sampling sites distribution.

Figure S2. Chromatogram (left) and mass spectrum (right) of 29 identified PACs.

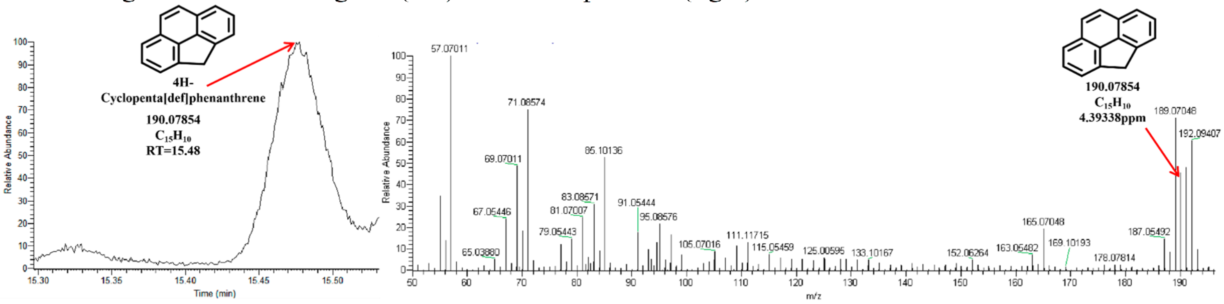

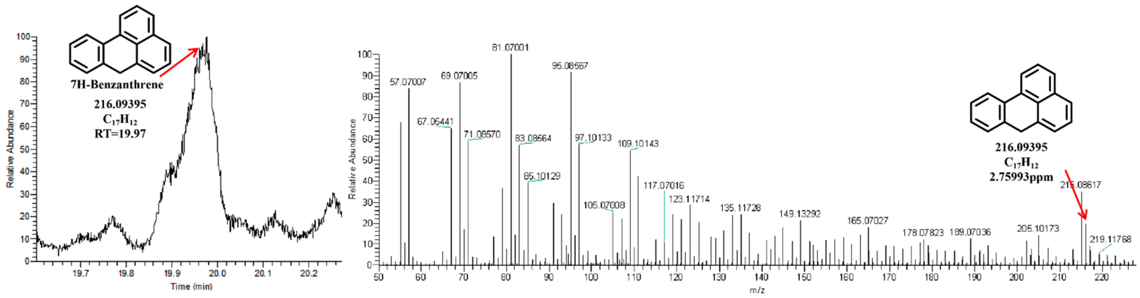

Figure S2-2. 7H-benzanthrene

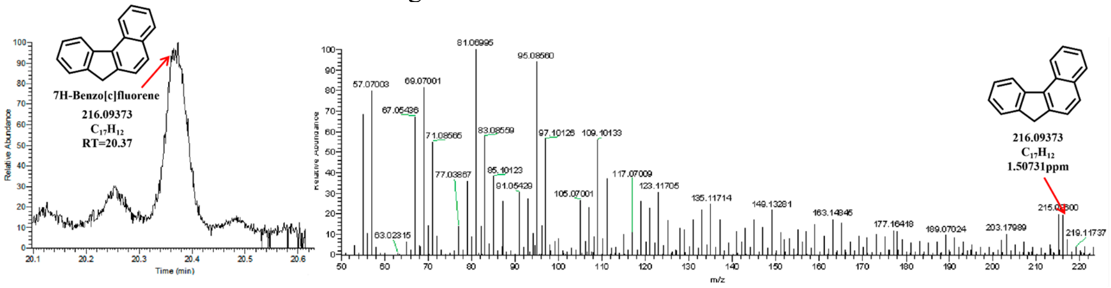

Figure S2-3. 7H-benzo[c]fluorene

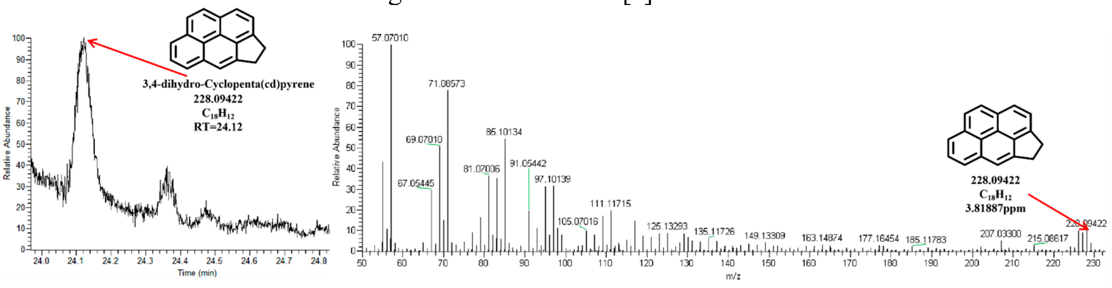

Figure S2-4. 3,4-dihydro-cyclopenta(cd)pyrene

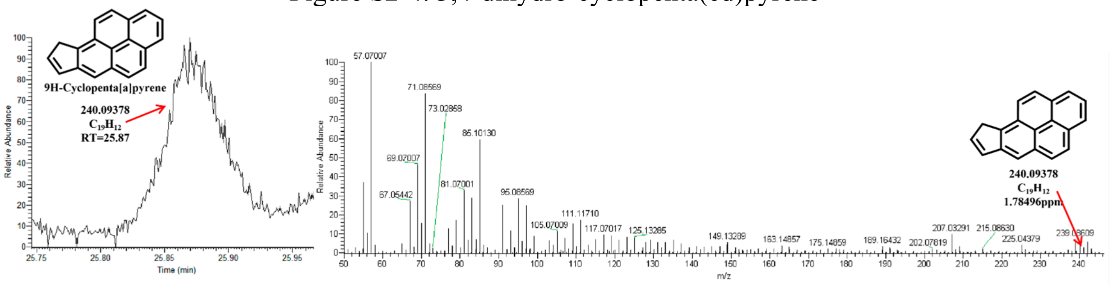

Figure S2-5. 9H-cyclopenta[a]pyrene

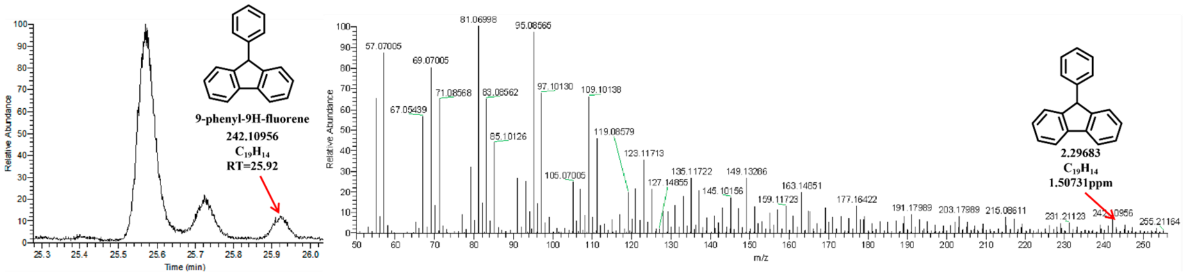

Figure S2-6. 9-phenyl-9H-fluorene

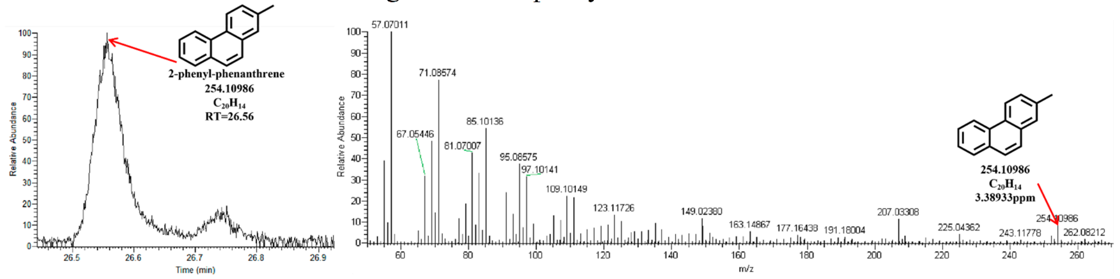

Figure S2-7. 2-phenyl-phenanthrene

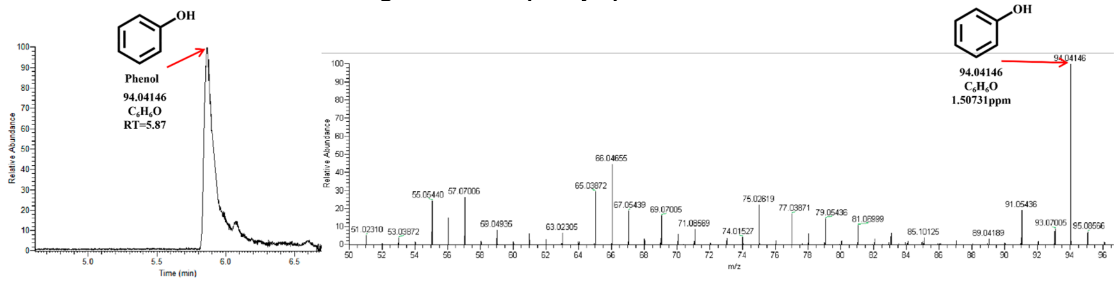

Figure S2-8. Phenol

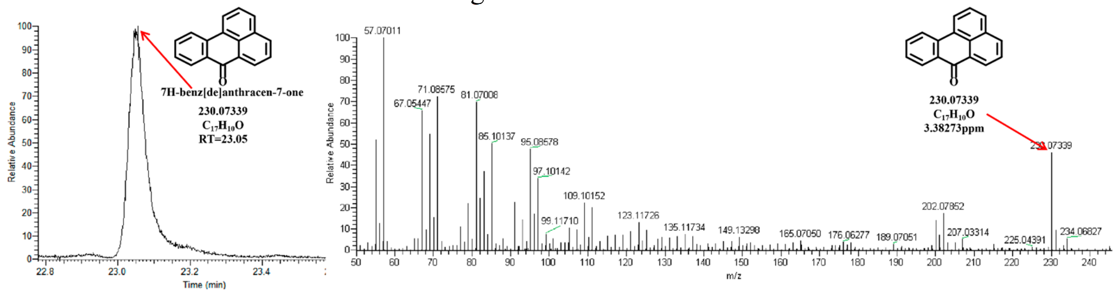

Figure S2-9. 7H-benz[de]anthracen-7-one

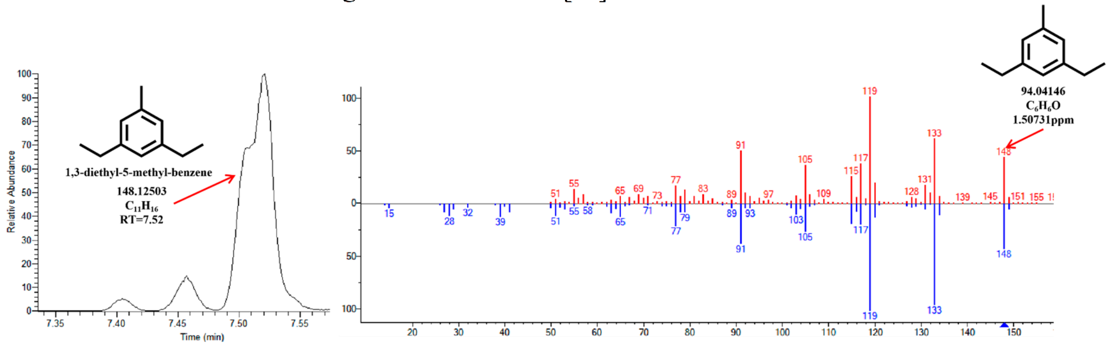

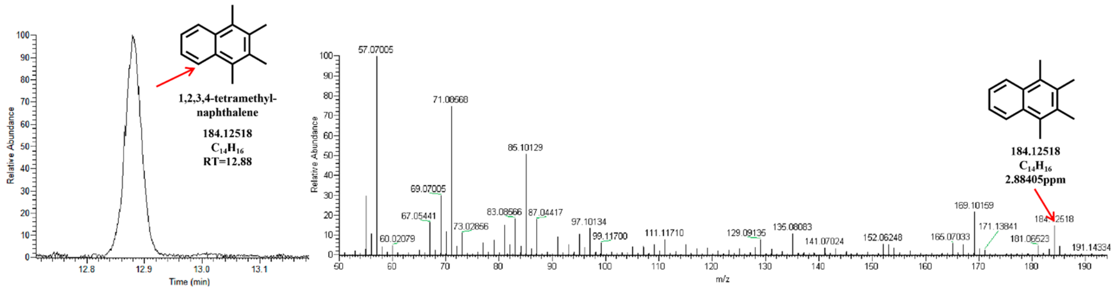

Figure S2-11. 1,2,3,4-tetramethyl-naphthalene

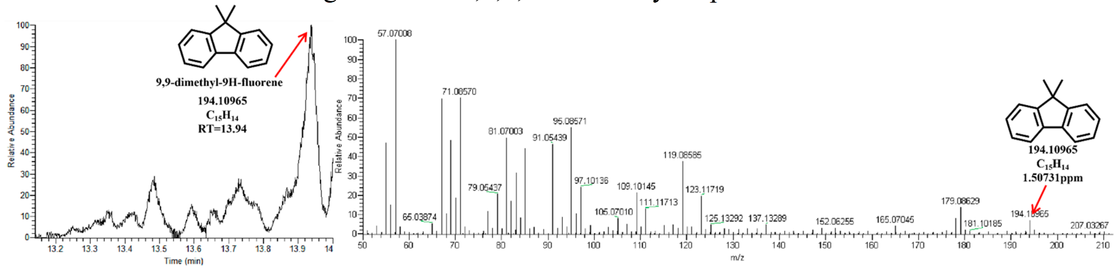

Figure S2-12. 9,9-dimethyl-9H-fluorene

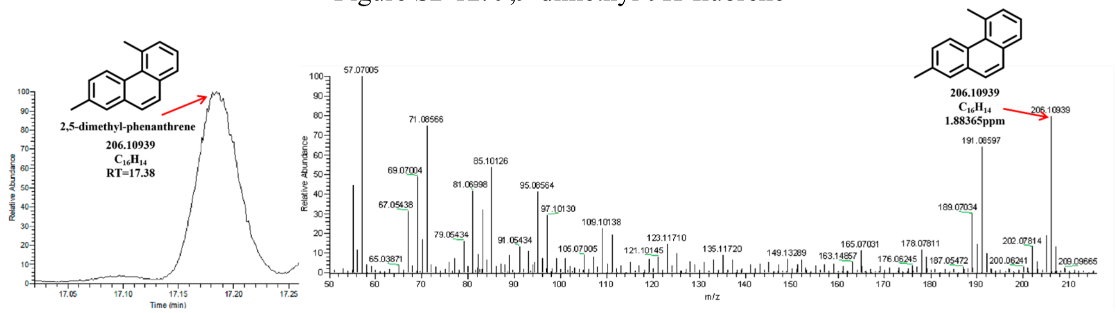

Figure S2-13. 2,5-dimethyl-phenanthrene

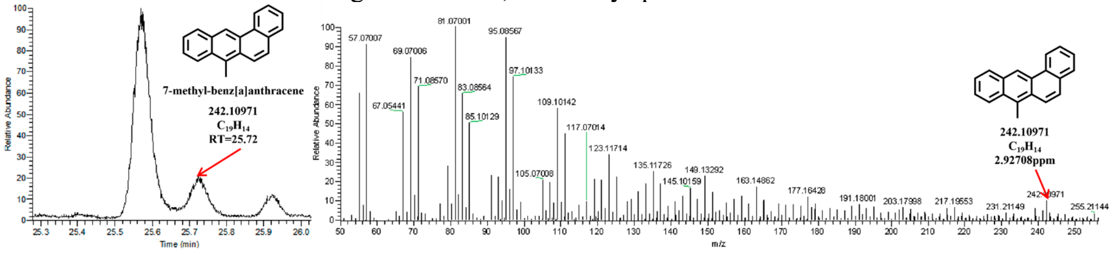

Figure S2-14. 7-methyl-benz[a]anthracene

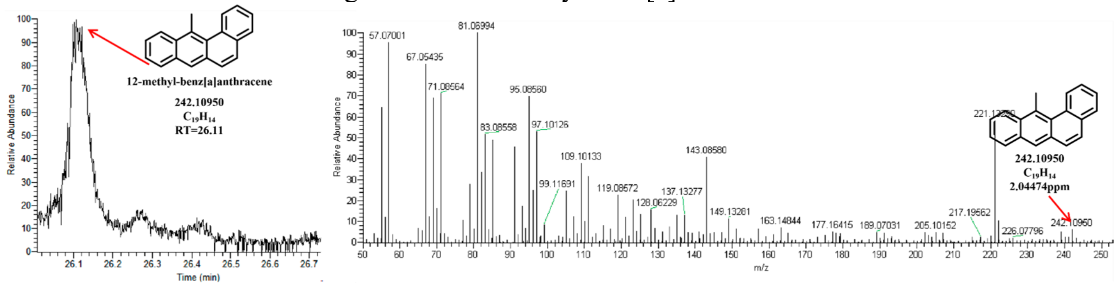

Figure S2-15. 12-methyl-benz[a]anthracene

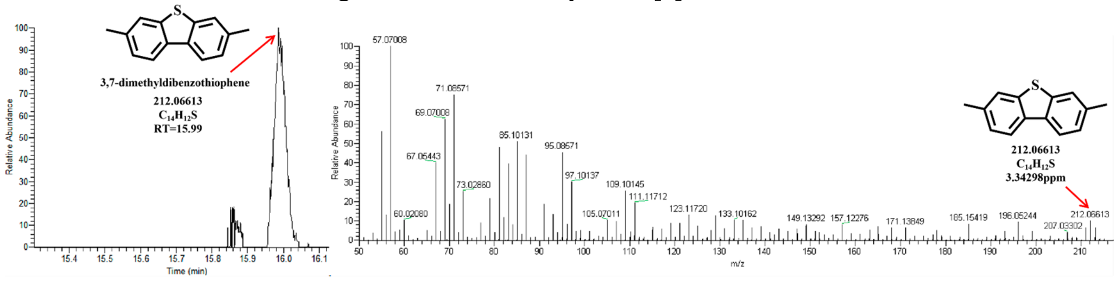

Figure S2-16. 3,7-dimethyldibenzothiophene

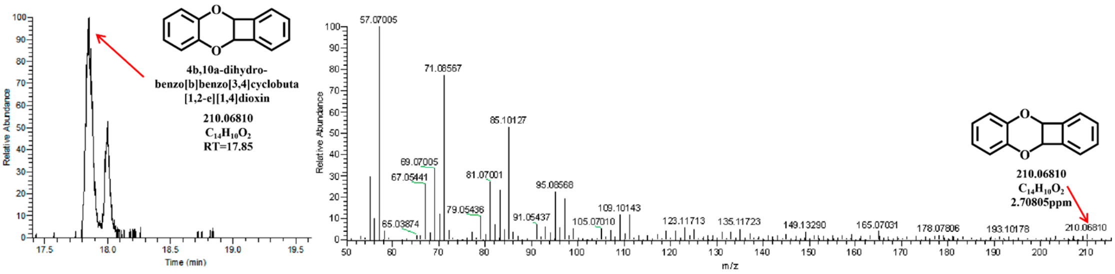

Figure S2-17. 4b,10a-dihydro-benzo[b]benzo[3,4]cyclobuta[1,2-e][1,4]dioxin

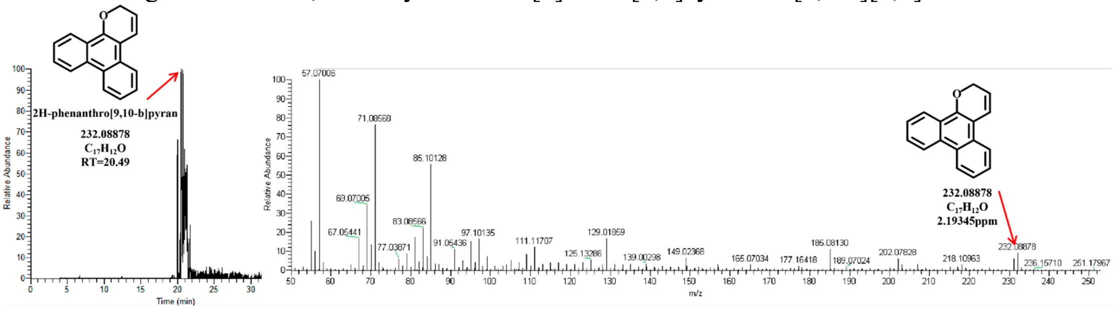

Figure S2-18. 2H-phenanthro[9,10-b]pyran

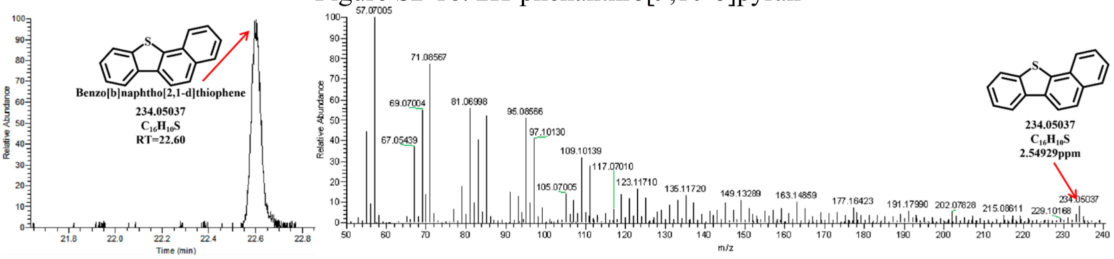

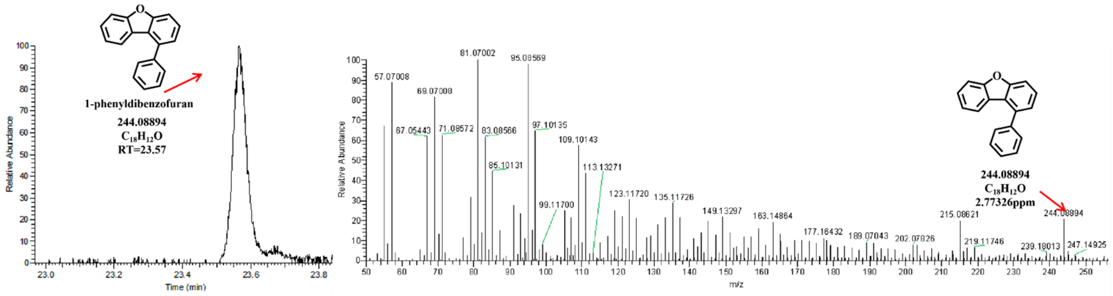

Figure S2-20. 1-phenyldibenzofuran

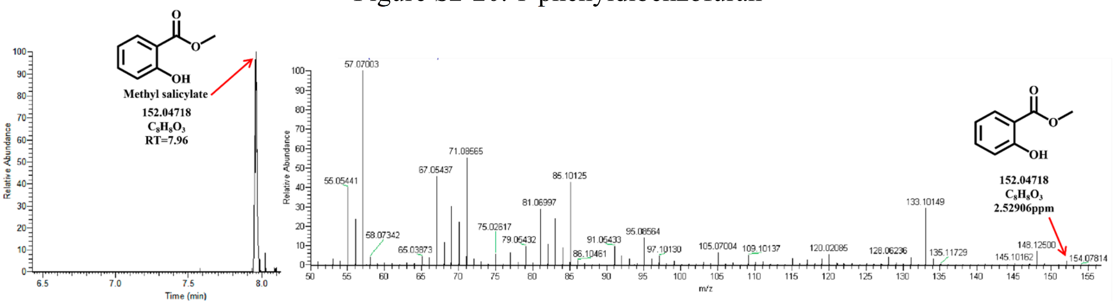

Figure S2-21. Methyl salicylate

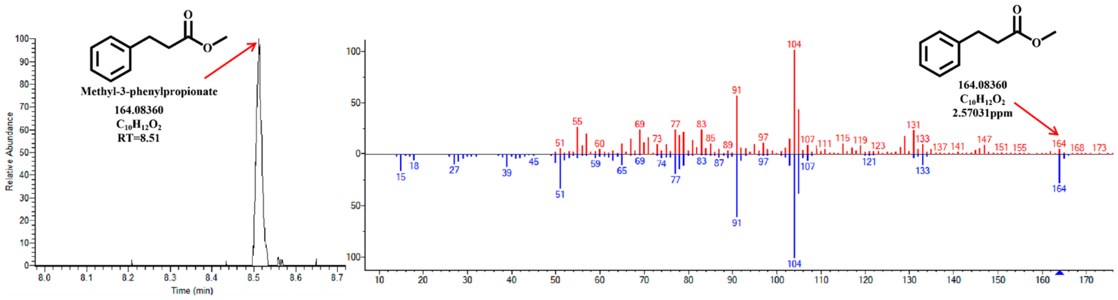

Figure S2-22. Methyl-3-phenylpropionate

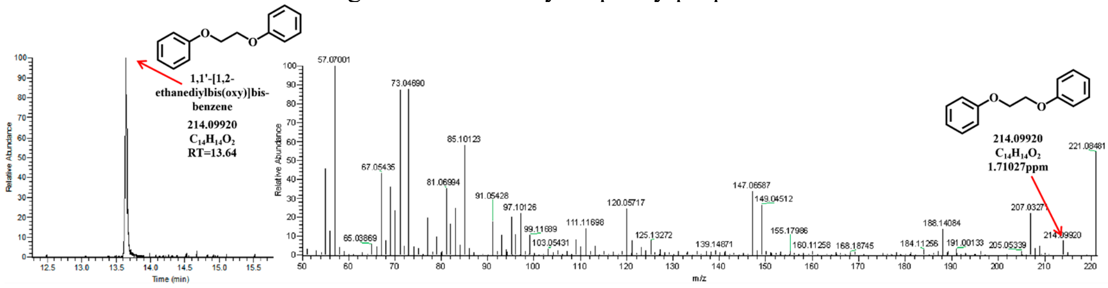

Figure S2-23. 1,1'-[1,2-ethanediylbis(oxy)]bis-benzene

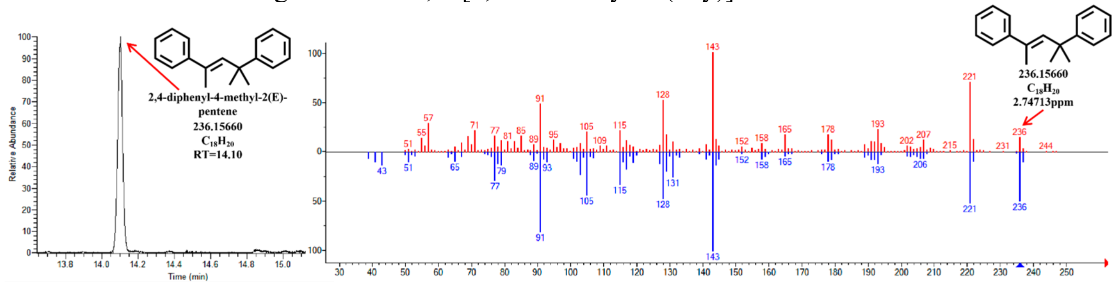

Figure S2-24. 2,4-diphenyl-4-methyl-2(E)-pentene

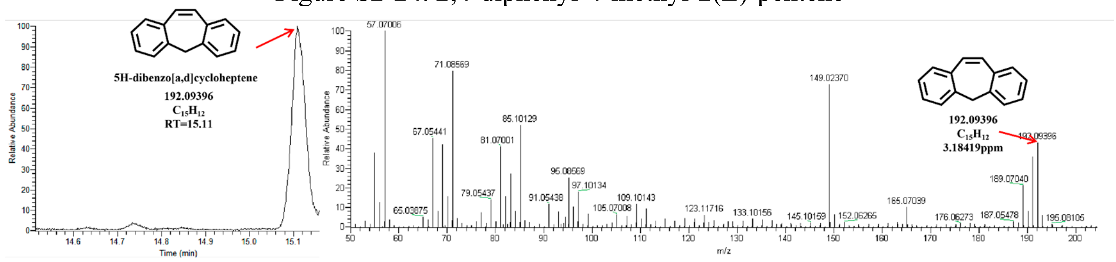

Figure S2-25. 5H-dibenzo[a,d]cycloheptene

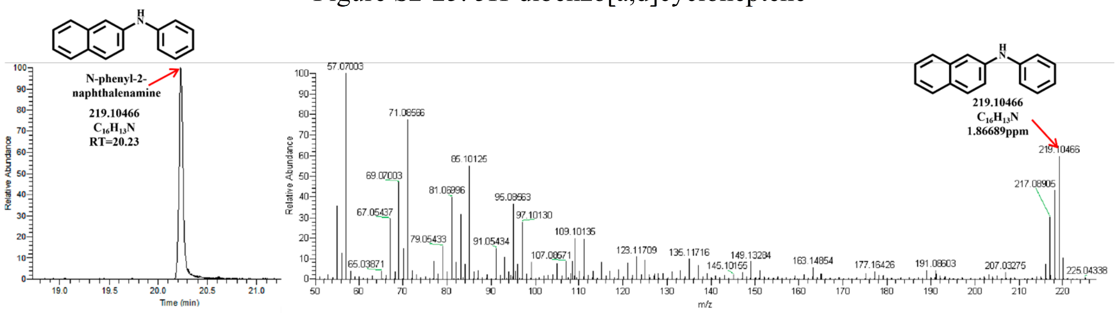

Figure S2-26. N-phenyl-2-naphthalenamine

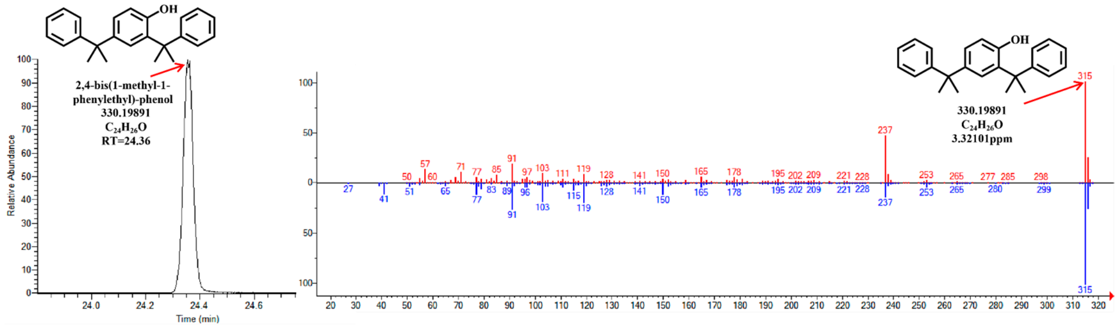

Figure S2-27. 2,4-bis(1-methyl-1-phenylethyl)-phenol

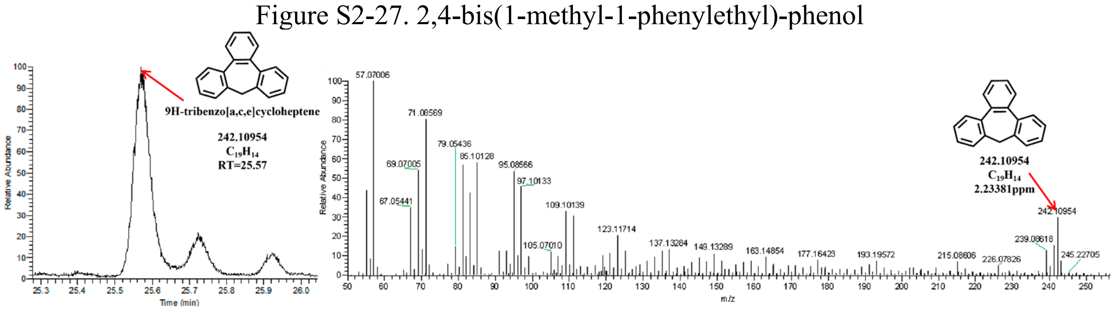

Figure S2-28. 9H-tribenzo[a,c,e]cycloheptene

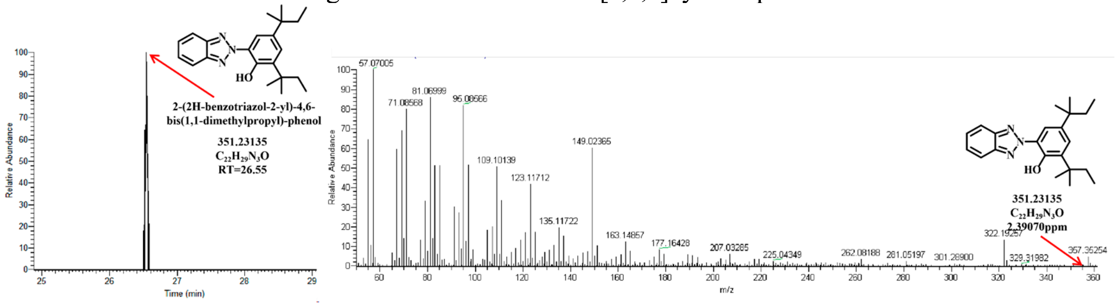

Figure S2-29. 2-(2H-benzotriazol-2-yl)-4,6-bis(1,1-dimethylpropyl)-phenol

References

1. Wang, L.; Wang, L.; Tao, W.; Smardon, R.C.; Shi, X.; Lu, X. Characteristics, sources, and health risk of polycyclic aromatic hydrocarbons in urban surface dust: a case study of the city of Xi'an in Northwest China. *Environmental Science and Pollution Research* **2016**, *23*, 13389-13402.
2. Yu, Y.; Wang, X.; Wang, B.; Tao, S.; Liu, W.; Wang, X.; Cao, J.; Li, B.; Lu, X.; Wong, M.H. Polycyclic aromatic hydrocarbon residues in human milk, placenta, and umbilical cord blood in Beijing, China. *Environmental science & technology* **2011**, *45*, 10235-10242.
3. Kong, J.; Yan, S.; Cao, X.; Zhang, Y.; Ran, C.; Chen, X.; Yang, S.; Li, S.; Zhang, L.; He, H. Quantitative source apportionment and health risk assessment for polycyclic aromatic hydrocarbon and their derivatives in indoor dust from housing and public buildings of a mega-city in China. *Journal of Hazardous Materials* **2025**, *486*, doi:10.1016/j.jhazmat.2024.137057.
4. Jiang, P.; Yang, L.; Chen, X.; Gao, Y.; Li, Y.; Zhang, J.; Zhao, T.; Yu, H.; Wang, W. Impact of dust storms on NPAHs and OPAHs in PM2. 5 in Jinan, China, in spring 2016: Concentrations, health risks, and sources. *Aerosol and Air Quality Research* **2018**, *18*, 471-484.
5. Ali, N. Polycyclic aromatic hydrocarbons (PAHs) in indoor air and dust samples of different Saudi microenvironments; health and carcinogenic risk assessment for the general population. *Science of the Total Environment* **2019**, *696*, 133995.
6. UNDP. International Human Development Index Ghana. **2012**.
7. Ferreira-Baptista, L.; Miguel, E.D. Geochemistry and risk assessment of street dust in Luanda, Angola: A tropical urban environment. *Atmospheric Environment* **2005**, *39*, 4501-4512.
8. U.S.EPA. Supplemental guidance for developing soil screening levels for superfund sites. Office of Solid Waste and Emergency Response (OSWER). **2001**.
9. Wei, C.; Bandowe, B.A.M.; Han, Y.; Cao, J.; Zhan, C.; Wilcke, W. Polycyclic aromatic hydrocarbons (PAHs) and their derivatives (alkyl-PAHs, oxygenated-PAHs, nitrated-PAHs and azaarenes) in urban road dusts from Xi'an, Central China. *Chemosphere* **2015**, *134*, 512-520.
10. Eilen A. Vik, G.B., Terje Farestveit (Grøner AS), and others. Guidelines for the risk assessment of contaminated sites. **1999**.
11. U.S.EPA. Exposure factors handbook. *Office of research and Development, Washington, DC* **2011**, *20460*, 2-6.
